# Cigarette Butts as an Emerging Urban Habitat Driving Microbial Niche Differentiation

**DOI:** 10.34133/research.1380

**Published:** 2026-07-27

**Authors:** Ting Xie, Jia-Yang Xu, Da Lin, Yang Liu, Yi-Fei Wang, Zhu-Gen Yang, Patrick K. H. Lee, Dong Zhu

**Affiliations:** ^1^ State Key Laboratory of Regional and Urban Ecology, Ningbo Urban Environment Observation and Research Station, Institute of Urban Environment, Chinese Academy of Sciences, Xiamen 361021, China.; ^2^ Zhejiang Key Laboratory of Pollution Control for Port-Petrochemical Industry, CAS Haixi Industrial Technology Innovation Center in Beilun, Ningbo 315830, China.; ^3^ University of Chinese Academy of Sciences, Beijing 100049, China.; ^4^ School of Marine Sciences, Ningbo University, Ningbo 315211, China.; ^5^ Faculty of Engineering and Applied Sciences, Cranfield University, Milton Keynes MK43 0AL, UK.; ^6^ School of Energy and Environment and State Key Laboratory of Marine Pollution, City University of Hong Kong, Hong Kong SAR, China.

## Abstract

Cigarette butts are common yet overlooked pollutants in urban environments. How this anthropogenic niche shapes microbial life-history strategies and evolutionary mechanisms remains poorly understood, limiting assessments of microbial adaption and urban ecosystem health. However, systematic and multiscale evidence on the ecological effects of cigarette butts on microbial communities remains scarce. Here, we collected cigarette butts, litter, and soil samples from urban parks in 35 Chinese cities and integrated third-generation 16S ribosomal RNA sequencing, metagenomics, and transcriptomics to resolve microbial community composition, functional potential, and evolutionary patterns. The results revealed that microbial communities in cigarette butts were shaped by strong deterministic processes, showed low spatial heterogeneity, and were taxonomically distinct from those in natural niche (i.e., litter) and surrounding soil, with notable enrichment of Proteobacteria, particularly the family Pseudomonadaceae. Functional trait analysis showed that butt-associated communities favored environmental responsiveness and fast-growth strategies, contrasting with metabolism- and resource acquisition-oriented strategies in the litter. Population genomic analysis suggested stronger positive selection in cigarette butt-associated Pseudomonadaceae, while the pure culture experiment provided strain-level evidence that cigarette butt exposure induced the up-regulation of key functional genes in *Pseudomonas aeruginosa* PAO1. This study demonstrates that cigarette butts, as an emerging ecological niche, reshape microbial community assembly, life-history strategies, and adaptive evolution, offering new insights into microbe-driven evolution on artificial surfaces.

## Introduction

Urbanization has led to the extensive accumulation of anthropogenic waste in city environments, giving rise to distinctive yet often overlooked ecological niches. Among these, cigarette butts represent one of the most ubiquitous forms of urban litter, with an estimated 5.7 trillion being discarded annually worldwide [1,2]. Cigarette butts, primarily composed of cellulose acetate, have been reported to contain and release a complex mixture of toxic compounds, including nicotine, polycyclic aromatic hydrocarbons, and heavy metals, which may pose environmental and public health risks [3]. Discarded cigarette butts are highly toxic to water environments [4,5] and aquatic organisms [3,6], and their chemical recalcitrance and environmental persistence suggest that they may serve as chronic sources of ecological disturbance. Previous studies have shown that artificial surfaces created by anthropogenic activities often constitute novel ecological niches for microbial colonization [7,8]. For example, artificial interfaces such as plastic interstices [7,9] as well as building surfaces [8,10] can support unique microbial communities under strong selective pressure. Green spaces in urban parks, which are common sites where cigarette butts are discarded and important interfaces between human activity and nature, provide ecologically relevant settings to investigate the responses of microbial communities to pollution. However, little is known about how this micropolluted anthropogenic niche influences the composition, ecological functions, and evolutionary traits of microbial communities it harbors, especially in comparison with natural niches. In this ecological context, soil serves as the environmental background, while the litter is considered a natural niche, and cigarette butts represent a typical anthropogenic niche. This framework provides a comparative approach to examine how cigarette butts serve as a selective niche for microbial colonization and functional adaptation in urban ecosystems.

Microorganisms exhibit distinct community structures and functional traits across different ecological niches [11,12], with their successional dynamics being generally shaped by the interplay between deterministic selection and stochastic drift [13,14]. In natural substrates such as soil and aquatic environments, the structure of microbial communities depends on a combination of resource availability, microhabitat heterogeneity, and stochastic colonization events, which results in high spatial heterogeneity [14–16]. In contrast, in environments subjected to long-term pollution stress, such as heavy metal-contaminated soils or antibiotic-polluted water bodies, there is a tendency toward stronger environmental filtering, leading to the dominance of deterministic processes [14,17–19], which determine the occupation of core ecological niches by microbial taxa with high stress resistance and specific metabolic capabilities [20–22]. However, whether cigarette butts act as distinct anthropogenic microhabitats that shape their associated microbial communities and may drive deterministic processes influencing microbial composition and function remains insufficiently explored. Recent studies have shown that such deterministic pressures not only shape the taxonomic composition of communities but also drive shifts in microbial ecological adaptation strategies and functional differentiation under varying environmental conditions. This differentiation can be systematically interpreted based on the trade-offs inherent in life-history strategies [23,24]. Life-history theory emphasizes the dynamic balance among microbial growth rate, resource acquisition capacity, and stress tolerance [25,26], providing a valuable framework for understanding microbial niche partitioning, community assembly, and evolutionary processes. For instance, in resource-poor and highly stressful environments such as barren soils, microbial communities often exhibit low ribosomal RNA (rRNA) gene copy numbers, slow growth rates, and weak codon usage bias, which are indicative of a conservative strategy focused on resource conservation and survival (oligotrophic, stress-tolerant) [27]. In contrast, in resource-enriched anaerobic environments such as those found at wastewater treatment facilities, dominant taxa typically possess smaller genomes, lower genomic guanine–cytosine (GC) content, and stronger codon usage bias, reflecting a competitive strategy adapted for rapid growth and resource exploitation (copiotrophic, competitive) [28]. These genomic functional traits reflect long-term adaptive trade-offs between resource acquisition and stress tolerance, offering theoretical support for understanding microbial niche differentiation and evolution from a trait-based perspective. However, it remains unclear whether cigarette butts, as an emerging urban niche, harbor microbial communities that exhibit life-history strategies distinct from those observed in natural environments such as soil or the litter. Based on the above studies, we hypothesized that cigarette butts undergo stronger environmental filtering, leading to specific microbial community assembly and life-history strategies distinct from those in natural niches (for example in the litter).

In addition to influencing microbial traits at the community level, environmental stress can drive rapid genomic diversification and elevated selection pressure within dominant taxa. Beyond life-history strategies, microbial genomic microdiversity provides another crucial perspective for understanding microbial adaptation. Characterized by single-nucleotide variations (SNVs) and signatures of positive selection on functional genes among other things, microbial genomic microdiversity is emerging as a powerful lens through which to investigate microbial evolutionary responses under pollution stress [29]. Previous studies have shown that environmental pollutants, including antibiotics [30] and heavy metals [31], as well as climate change (e.g., global warming) [32], can drive adaptive evolution in genes related to antibiotic resistance, motility, and pathogenicity. However, it remains largely unexplored whether emerging pollutants in urban environments, such as cigarette butts, can also trigger adaptive evolution in key functional genes. Therefore, we propose a second hypothesis: Cigarette butts, as an anthropogenic niche, may enhance selective pressures, driving genomic microdiversity shifts in core microbial taxa and accelerating the adaptive evolution of key functional genes, thus increasing microbial adaptation to cigarette butt-induced stress.

In the present study, we systematically assessed the potential of cigarette butts (as an anthropogenic niche) to reshape the structure and function of microbial communities harbored on their surfaces by integrating long-read 16S rRNA gene sequencing, metagenomic sequencing, trait-based ecological analyses, and laboratory incubation experiments. We comprehensively investigated microbial communities in cigarette butts collected from 35 urban parks in China and compared them with those in soil, used as the environmental background, and the litter, considered a natural niche, to evaluate the differences. Our objectives were to (a) evaluate whether cigarette butts represent a unique ecological niche capable of restructuring microbial community assembly and life-history strategies; and (b) determine whether cigarette butts drive adaptive evolution in dominant microbial taxa, especially in genes associated with key ecological functions. By integrating community ecology approaches and evolutionary genomics, this study provides novel insights into how anthropogenic pollutants reshape microbial ecosystems, highlights the potential role of cigarette butts as emerging hotspots of urban microevolution, and advances our understanding of how human activities drive microbial evolution and environmental adaptation.

## Results

### Bacterial community assembly shows unique patterns across ecological niches

**Fig. 1.**
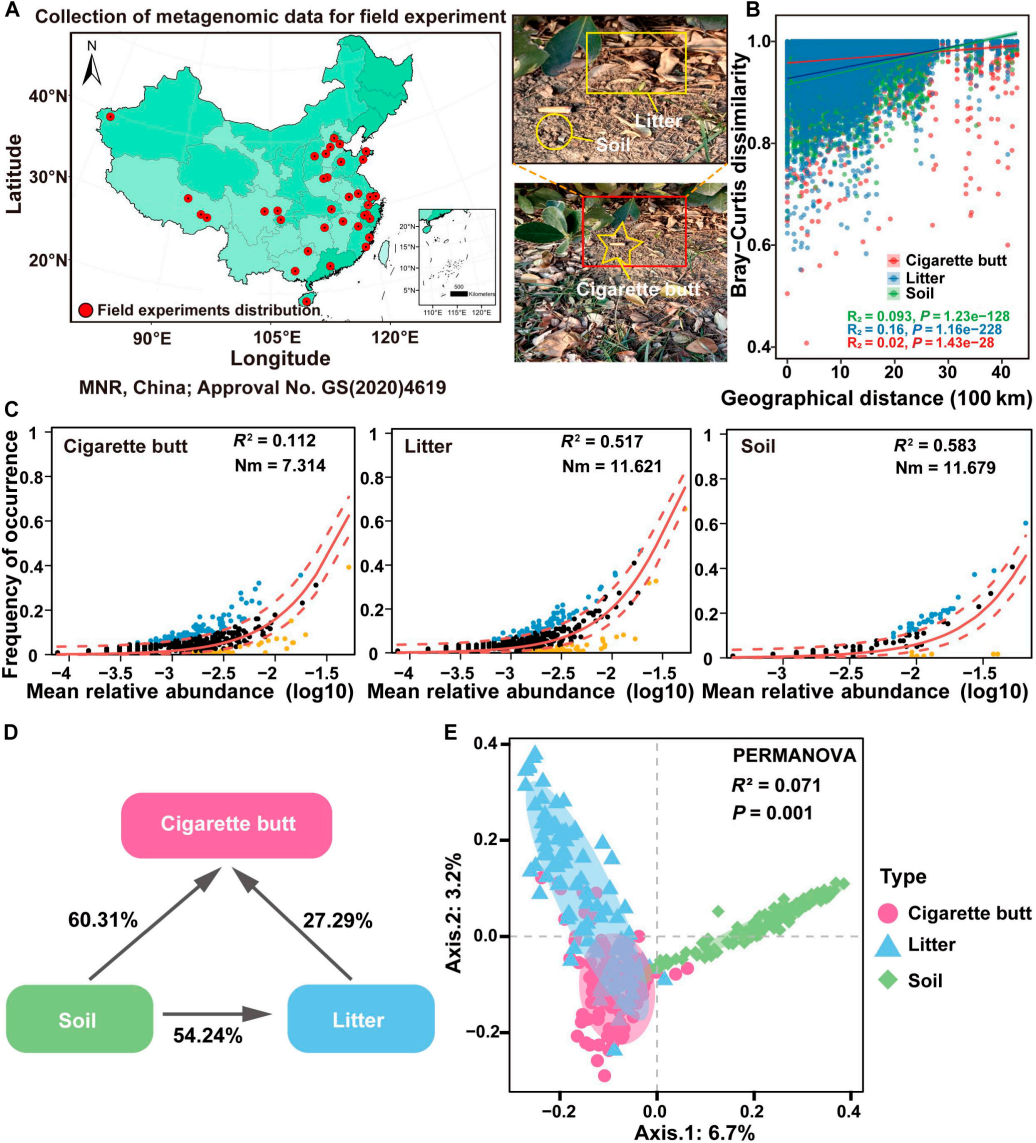
Geographic patterns, assembly mechanisms, and cross-habitat linkages of bacterial communities in cigarette butts, the litter, and soil. (A) Geographic distribution of sampling sites across urban greenspaces in China. Red dots indicate the sampling locations. The base map was derived from the standard map supervised by the Ministry of Natural Resources of China (MNR), Approval No. GS(2020)4619, with no modification to the boundary. In the photographs, the yellow circle, the yellow rectangle, and the yellow star indicate the sampling points for the soil, litter, and cigarette butts, respectively. (B) Distance–decay relationships illustrating Bray–Curtis dissimilarities of bacterial communities against geographic distance across different sample types, with fitted regression lines (solid) and corresponding statistical significance (*R*^2^ and *P* values). (C) Analyses of bacterial communities using the neutral community model (NCM). Solid red lines represent the optimal NCM fit, dashed lines indicate the 95% confidence intervals, and colored points indicate bacteria occurring more frequently (above neutral, blue), less frequently (below neutral, orange), or as frequently as predicted (neutral, gray). Parameter immigration rates (Nm) and *R*^2^ reflect the immigration rate and NCM fit, respectively. (D) Source–sink relationships and cross-habitat contributions estimated by fast expectation-maximization microbial source tracking; percentages indicate the proportion each source contributes to its corresponding sink community. (E) Principal coordinates analysis showing differences in bacterial community composition among cigarette butt, litter, and soil samples based on Bray–Curtis dissimilarities. Permutational multivariate analysis of variance (PERMANOVA) results are shown in the panel.

To examine bacterial community patterns within the broader soil environment, the communities within the litter (natural niche) were compared with those associated with cigarette butts (artificial niche) in greenspaces from 35 urban parks in China (Fig. 1A). The bacterial community composition in all niches was dominated by Proteobacteria at the phylum level, which accounted for 79.7% to 89.3% of the total relative abundance (Fig. S1A). At the family level, Proteobacteria enriched in the 3 ecological niches were dominated by Acetobacteraceae and Pseudomonadaceae (Fig. S1A). Notably, cigarette butts exhibited the highest relative abundances of Proteobacteria and Pseudomonadaceae, with the distribution patterns of these 2 taxa significantly differing from those observed in the litter and soil niches (*P* < 0.05) (Fig. S1B). In line with this finding, fast expectation-maximization microbial source tracking revealed strong cross-habitat contributions (Fig. 1D), with soil contributing 60.3% and 54.2% to the communities in cigarette butts and the litter, respectively, and the litter contributing only 27.3% to the communities in cigarette butts. Principal coordinates analysis (PCoA) further showed that the bacterial community structure in cigarette butts was distinctively different from that in the litter and soil, with taxa from cigarette butts clustering closely together (Fig. 1E; permutational multivariate analysis of variance, *R*^2^ = 0.071, *P* = 0.001).

Distance–decay relationships (DDRs) for bacterial community dissimilarity showed significant correlations with geographic distance (*P* < 0.01; Fig. 1B). Among the 3 ecological niches examined, the litter exhibited the steepest DDR slope, followed by soil, and the lowest value was observed for cigarette butts, indicating that the latter niche had the weakest distance–decay effect on bacterial community dissimilarity. These results suggest that the bacterial communities across different ecological niches are characterized by distinct biogeographic patterns.

We further investigated the potential factors contributing to differences among bacterial communities by applying the neutral community model (NCM) to evaluate the role of neutral processes in bacterial community assembly across ecological niches. As shown in Fig. 1C, cigarette butts exhibited the lowest NCM fit (*R*^2^ = 0.11), whereas soil (*R*^2^ = 0.58) and litter (*R*^2^ = 0.52) showed relatively high fits. These results suggest that bacterial community assembly in cigarette butts was less consistent with neutral processes and may be more strongly influenced by deterministic selection than that in soil and litter. Moreover, infer Community Assembly Mechanisms by Phylogenetic-bin-based null model analysis showed that the relative importance of deterministic processes differed significantly among niches (Kruskal–Wallis, *P* = 0.027; Fig. S2A). Cigarette butts exhibited a higher deterministic contribution than soil and litter, increasing by approximately 11.2% to 69.1%, whereas their stochastic contribution was relatively lower (Kruskal–Wallis, *P* = 0.027; Fig. S2C). Homogeneous selection (HoS) further dominated the deterministic component across all niches (Fig. S2B), suggesting that the relatively stronger deterministic assembly in cigarette butts was mainly attributable to HoS. In contrast, stochastic processes were mainly driven by dispersal limitation, especially in soil and litter communities where stochastic assembly made a relatively larger contribution [33] (Fig. S2D). This underscores the distinct role of cigarette butts as a unique microecological niche within urban environments.

### Unique functional traits in bacterial communities within cigarette butts

**Fig. 2.**
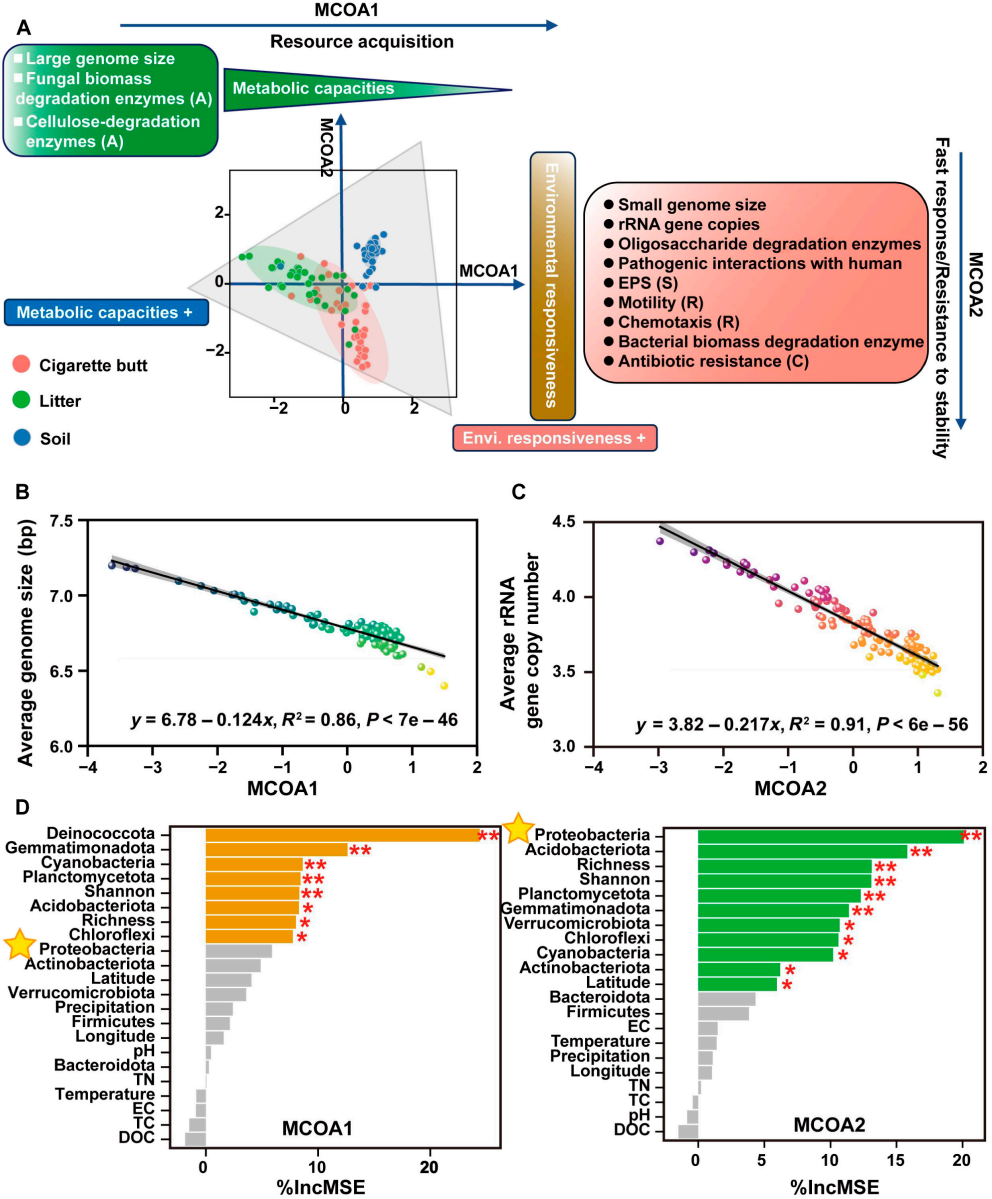
Trait dimensions and functional strategies of bacterial communities in cigarette butt, litter, and soil samples. (A) Two-dimensional trait space from multitable co-inertia analysis (MCOA) depicting the associations among bacterial metagenomic traits across cigarette butt, litter, and soil samples (*n* = 105). In the trait lists, the letters in parentheses indicate the links between traits and the CSR (competitor, stress-tolerant, ruderal) and YAS (high-yield, resource acquisition, stress tolerance) strategies previously made in theoretical studies (Data S1). Arrows indicate the directions of major life-history strategies, including resource acquisition and environmental responsiveness, inferred from trait loadings. (B and C) Correlations between MCOA dimensions and average genome size (bp) (B) or average 16S ribosomal RNA (rRNA) gene copy number (C). Regression lines (solid) with 95% confidence intervals (shading) are shown, with *P* values indicating the significance of the regression slope (*t* test). (D) Relative importance of environmental factors and bacterial communities in predicting MCOA dimensions 1 and 2 based on random forest models. The importance values are shown as mean decrease in mean square error (%IncMSE). Bar colors correspond to the direction of the correlation with the MCOA dimensions. Orange and green bars represent positive correlations with MCOA1 and MCOA2, respectively, while gray bars indicate weak or no correlation. Asterisks denote statistical significance (*P < 0.05, **P < 0.01).

Using multitable co-inertia analysis (MCOA), we identified 2 major dimensions that captured most of the variation in metagenomic community-aggregated traits. The first trait dimension (MCOA1) explained 71.1% of the variation in functional traits associated with bacterial life-history strategies, whereas the second dimension (MCOA2) accounted for 15.9% of this variation (Fig. S3A and B). Relative to the surrounding soil, the bacterial communities within cigarette butts (artificial niche) and litter (natural niche) exhibited distinct distributions along these 2 trait dimensions (Fig. 2A). The lower end of MCOA1, primarily represented by bacterial communities within the litter, was associated with larger average genome sizes and fungal biomass degradation enzymes, reflecting a more complex capacity for metabolizing organic matter. These communities were enriched in enzymes capable of breaking down complex polysaccharides from fungi and plants, including oligosaccharide- and cellulose-degrading enzymes from the Carbohydrate-Active enZymes (CAZy) family, such as GH1, GH76, GH12, and GH18 (Fig. S3A). Consistently, the total abundance of CAZy genes was significantly higher in the microbiome of the litter than in that of cigarette butts and soil, underscoring its enhanced carbohydrate metabolic potential (*P* < 0.001; Fig. S4). Thus, the lower end of MCOA1 was defined by bacterial communities with larger genomes and more complex metabolic and resource acquisition strategies.

In contrast, the lower end of MCOA2 was dominated by bacterial communities from cigarette butts, exhibiting traits of smaller genome sizes, environmental responsiveness, and rapid proliferation (Fig. 2A). These communities harbored genomic features associated with fast growth (e.g., high number of 16S rRNA gene copies), motility, chemotaxis, bacterial biomass and oligosaccharide degradation, resistance to environmental stressors (e.g., pathogenic interactions with humans and antibiotic resistance), and exopolysaccharide (EPS) biosynthesis (Fig. S3B). In line with this, the 16S rRNA gene copy number was significantly higher in the communities within cigarette butts than in those in the litter and soil (*P* < 0.001; Fig. S5A). In contrast, average genome size was largest and smallest in the litter and cigarette butt communities, respectively (*P* < 0.001; Fig. S5B). The soil communities exhibited the highest GC content, followed by the cigarette butt and litter communities. (Fig. S5C). Linear regression analysis further demonstrated that MCOA1 was significantly negatively correlated with average genome size (*R^2^* = 0.86, *P* < 0.001) (Fig. 2B), whereas MCOA2 exhibited a strong negative correlation with 16S rRNA copy number (*R*^2^ = 0.91, *P* < 0.001) (Fig. 2C). Overall, MCOA1 primarily reflected gradients in genome size and metabolic capacity, whereas MCOA2 represented variation related to environmental responsiveness and rapid adaptation traits.

Random forest analyses further identified the key drivers shaping the distribution patterns of the trait dimensions (MCOA1 and MCOA2) in bacterial communities (Fig. 2D). The composition and diversity (e.g., the Shannon index and richness indexes) of communities were the main factors influencing both dimensions, with significant differences detected in their response patterns to these dimensions. The abundance of Proteobacteria was the strongest predictor of MCOA2, whereas it had no significant effect on MCOA1. In contrast, Acidobacteriota and Planctomycetota were the main drivers of MCOA1.

**Fig. 3.**
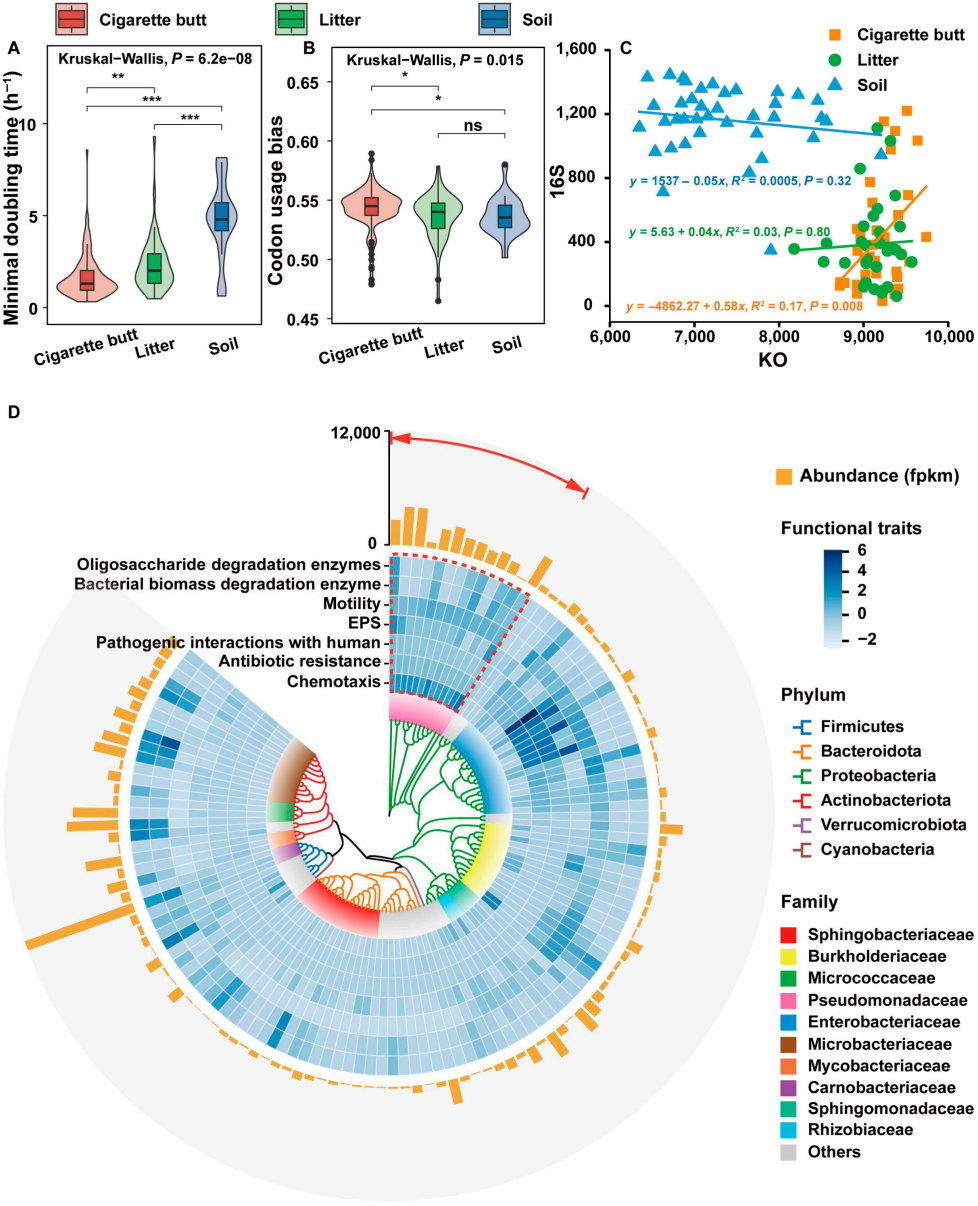
Distribution patterns of bacterial taxa and functional traits derived from high-quality bacterial metagenome-assembled genomes (MAGs). (A) Minimum doubling time and (B) codon usage bias of MAGs across cigarette butts, litter, and soil. (C) Linear regression analysis of bacterial taxonomic richness (16S) and functional gene richness (KO). (D) Characteristics of bacterial MAGs associated with cigarette butts, including relative abundance (*z*-score normalized), maximum growth rate, and the distribution of unique life-history traits within the cigarette butt microbiome.

Using metagenome assemblies, we recovered nonredundant, high-quality bacterial metagenome-assembled genomes (MAGs) (completeness > 90%, contamination < 5%) from cigarette butt, litter, and soil samples (Data S1). The bacterial communities from cigarette butts exhibited significantly shorter minimal doubling times and higher codon usage biases compared to those from the litter and soil (*P* < 0.05; Fig. 3A and B). Furthermore, a significant positive correlation between functional gene richness and bacterial taxonomy was observed only in cigarette butts (*R*^2^ = 0.17, *P* < 0.01), indicating a tight coupling of community composition and functional potential, whereas no such relationship was detected in the other 2 niches (Fig. 3C).

To further elucidate the ecological adaptive traits of bacterial communities within the cigarette butt microenvironment, we analyzed variations in life-history traits of bacteria from high-quality MAGs in cigarette butt samples to reveal bacterial adaptive strategies unique to this niche. The results revealed significant differences in both taxonomic composition and functional traits among cigarette butt-associated MAGs (Fig. 3D). In particular, the abundant Proteobacteria phylum found in cigarette butts exhibited clear advantages in chemotaxis, antibiotic resistance, pathogenic interactions with humans, EPS biosynthesis, motility, and the capacity to degrade bacterial biomass and oligosaccharides. Moreover, these specific life-history traits were mainly found in Pseudomonadaceae members, which represented the most abundant family in the cigarette butt microbiome, accounting for 21.6% of the total bacterial community (Fig. 3D and Fig. S6). Overall, these results highlight the pivotal role of Pseudomonadaceae in the specialized life-history strategies characteristic of cigarette butt microbiomes. Such taxonomic and functional differentiation is likely to be closely associated with anthropogenic chemical stressors and bacterial evolutionary processes in the cigarette butt environment.

### *Pseudomonas* species drive microdiversity in the cigarette butt microbiome

**Fig. 4.**
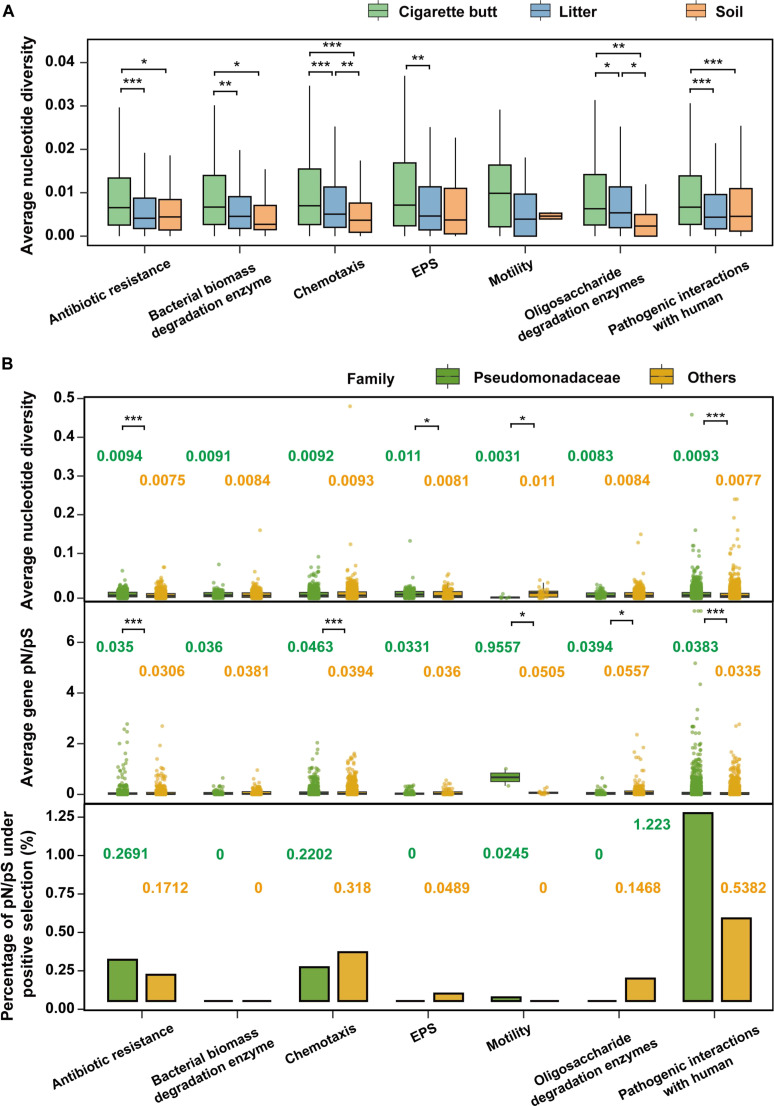
Microdiversity and evolutionary forces underlying distinct functional strategies in bacterial genomes associated with cigarette butts. (A) Average nucleotide diversity of genes involved in key ecological functions (e.g., antibiotic resistance, bacterial biomass and oligosaccharide degradation, chemotaxis, exopolysaccharide [EPS] biosynthesis, motility, and pathogenic interactions with humans) across cigarette butts, litter, and soil; (B) Comparison of average nucleotide diversity, average gene pN/pS, and proportion of genes under positive selection (pN/pS > 1) between Pseudomonadaceae and other bacterial families within each functional category in cigarette butt-associated communities.

Microdiversity in the cigarette butt, litter, and soil niches was examined to assess genetic differentiation among bacterial communities. Significant differences in evolutionary characteristics were observed across niches (*P* < 0.001). Notably, the bacterial community associated with cigarette butts exhibited the highest nucleotide diversity (0.008), SNV counts (289,858 bp), and number of both synonymous (234,757 bp) and nonsynonymous mutations (55,100 bp), followed by the communities in the litter and soil (Figs. S7A and S8). This pattern was also reflected in both the ratio of nonsynonymous to synonymous mutations (pN/pS) and the proportion of genes under positive selection (pN/pS > 1) (Fig. S7B and C), indicating a higher level of genomic variation and adaptive evolution in cigarette butt-associated bacterial communities. Moreover, these communities exhibited a significantly higher nucleotide diversity in life-history-related genes enriched in the cigarette butt environment, including genes regulating antibiotic resistance, chemotaxis, motility, EPS biosynthesis, human pathogenicity, bacterial biomass and oligosaccharide degradation, compared with the communities in the litter and soil (Fig. 4A; *P* < 0.001). In line with this finding, the genomes of cigarette butt-associated bacteria were shown to accumulate substantially higher SNVs in these functional categories, including both synonymous and nonsynonymous mutations, with particularly pronounced increases in the number of genes related to antibiotic resistance, chemotaxis, and human pathogenicity (Fig. S9).

To further elucidate the taxonomic distribution of this mutational pattern, we compared microdiversity metrics between Proteobacteria and all the other phyla and between Pseudomonadaceae and all the other families. Proteobacteria exhibited a significantly higher proportion of positively selected genes (1.1%, *P* < 0.05; Fig. S7D) and greater nucleotide diversity than other phyla (Fig. S10). These differences were particularly evident in life-history-related genes enriched in the cigarette butt environment, including genes regulating antibiotic resistance, chemotaxis, EPS biosynthesis, human pathogenicity, and oligosaccharide degradation (*P* < 0.001; Fig. S10). Furthermore, Proteobacteria displayed stronger signals of positive selection, especially for genes related to human pathogenicity (1.7%) and antibiotic resistance (0.4%) (Fig. S11). This phylum accumulated more SNVs across the abovementioned functional genes, with pronounced increases observed in genes regulating chemotaxis, EPS biosynthesis, and pathogenicity, showing consistent trends in both synonymous and nonsynonymous mutations (Fig. S12). At the family level, Pseudomonadaceae exhibited a significantly higher proportion of positively selected genes than other families (0.8%, *P* < 0.001; Fig. S7E). This family also showed significantly higher nucleotide diversity across major functional genes, such as genes regulating antibiotic resistance, EPS biosynthesis, biomass degradation, and pathogenicity (*P* < 0.05; Fig. 4B). Moreover, it displayed elevated pN/pS ratios and a higher proportion of genes under positive selection, particularly genes regulating antibiotic resistance, motility and pathogenicity, along with higher SNV accumulation in these functional genes (Fig. 4B and Fig. S13). In summary, Proteobacteria, and the Pseudomonadaceae family in particular, play a pivotal role in driving genetic variation and adaptive functional potential within cigarette butt-associated microbiomes.

### Exposure to cigarette butts enhances adaptive function in *P. aeruginosa*

To confirm the potential mechanisms underlying the functional responses of Pseudomonadaceae under exposure to cigarette butts, we conducted incubation experiments using *P. aeruginosa*, a model strain widely distributed in the environment and commonly used for studies on adaptive evolution. Three culture conditions, i.e., *P. aeruginosa* incubated with cigarette butts (PC, anthropogenic surface), wooden sticks (PW, natural substrate control), and glass rods (PG, inert artificial surface control), were established to distinguish effects specifically associated with cigarette butt exposure from natural substrate and inert surface effects.

**Fig. 5.**
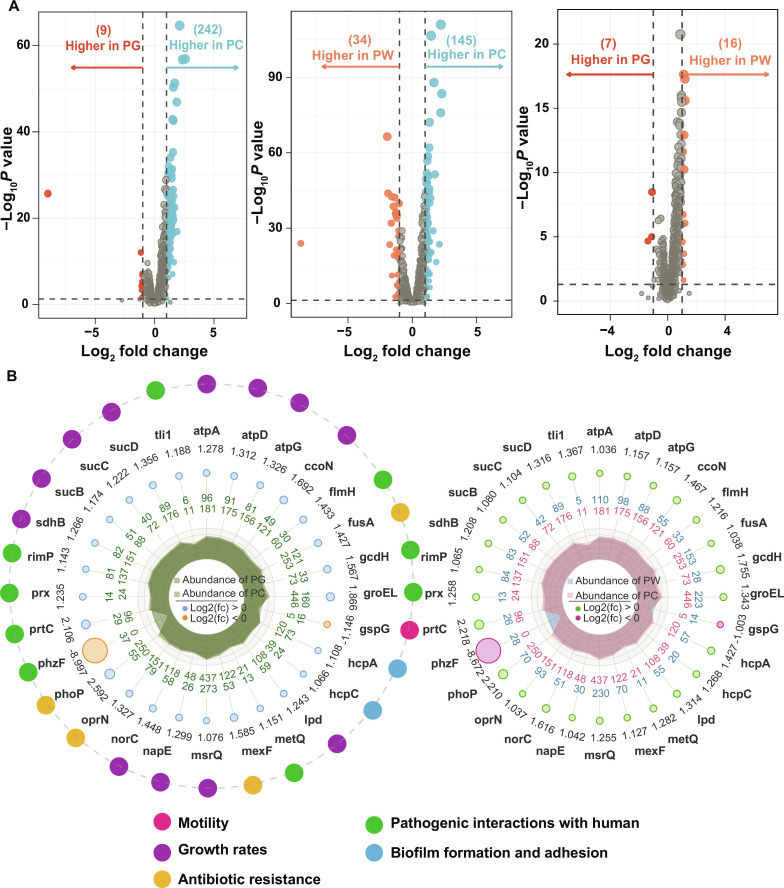
Functional enhancement of the model strain *Pseudomonas aeruginosa* exposed to cigarette butts for 30 d. (A) Volcano plots showing differentially expressed genes among 3 treatments: PG (*P. aeruginosa* incubated with glass rods), PW (*P. aeruginosa* incubated with wooden sticks), and PC (*P. aeruginosa* incubated with cigarette butts). Red, orange, and blue dots represent genes that are significantly differentially expressed among groups (|log₂FC| > 1, *P* < 0.05). In each volcano plot, genes on the right-hand side (log_2_FC > 1) were more highly expressed in the treatment indicated on the right, whereas genes on the left-hand side (log_2_FC < −1) were more highly expressed in the treatment indicated on the left. Specifically, the blue, orange, and red colors indicate highly expressed genes under PC, PW, and PG treatments, respectively. (B) Functional classification and abundance ratios of significantly enriched genes between PC and PG (left) and between PC and PW (right). Radial plots illustrate genes associated with motility (purple), growth rate (pink), biofilm formation and adhesion (blue), pathogenic interactions with humans (green), and antibiotic resistance (yellow). The outer circles denote log_2_ fold change, and inner values represent abundance ratios.

Exposure to cigarette butts was shown to significantly reshape the functional profile of *P. aeruginosa*. Transcriptome analysis revealed that the PC culture exhibited more differentially expressed genes (DEGs; 9 downregulated versus 242 up-regulated, for a total of 251, *P <* 0.05; Fig. 5A) compared with the PG culture. Similarly, 145 DEGs were up-regulated in PC, whereas only 34 were up-regulated in PW. In contrast, fewer DEGs were detected between PG and PW (16 up-regulated in PW and 7 up-regulated in PG). Together, these pairwise comparisons indicate that cigarette butt exposure elicited a distinct and more extensive transcriptional response in *P. aeruginosa* PAO1 than the natural substrate or inert artificial surface controls. A total of 28 DEGs between PC and PG were associated with motility (e.g., *gspG*), growth rate (e.g., *sucC* and *atpD*), biofilm formation and adhesion (e.g., *hcpA* and *hcpC*), pathogenic interactions with humans (e.g., *tli1* and *phzF*), and antibiotic resistance (e.g., *phoP*, *fusA*, and *mexF*) (Fig. 5B). Among these 28 genes, 26 (i.e., 92.9%) were up-regulated. Similar patterns of differential expression were also observed between PC and PW. These findings suggest that exposure to cigarette butts enhances adaptive traits in *P. aeruginosa* through widespread transcriptional reprogramming.

## Discussion

**Fig. 6.**
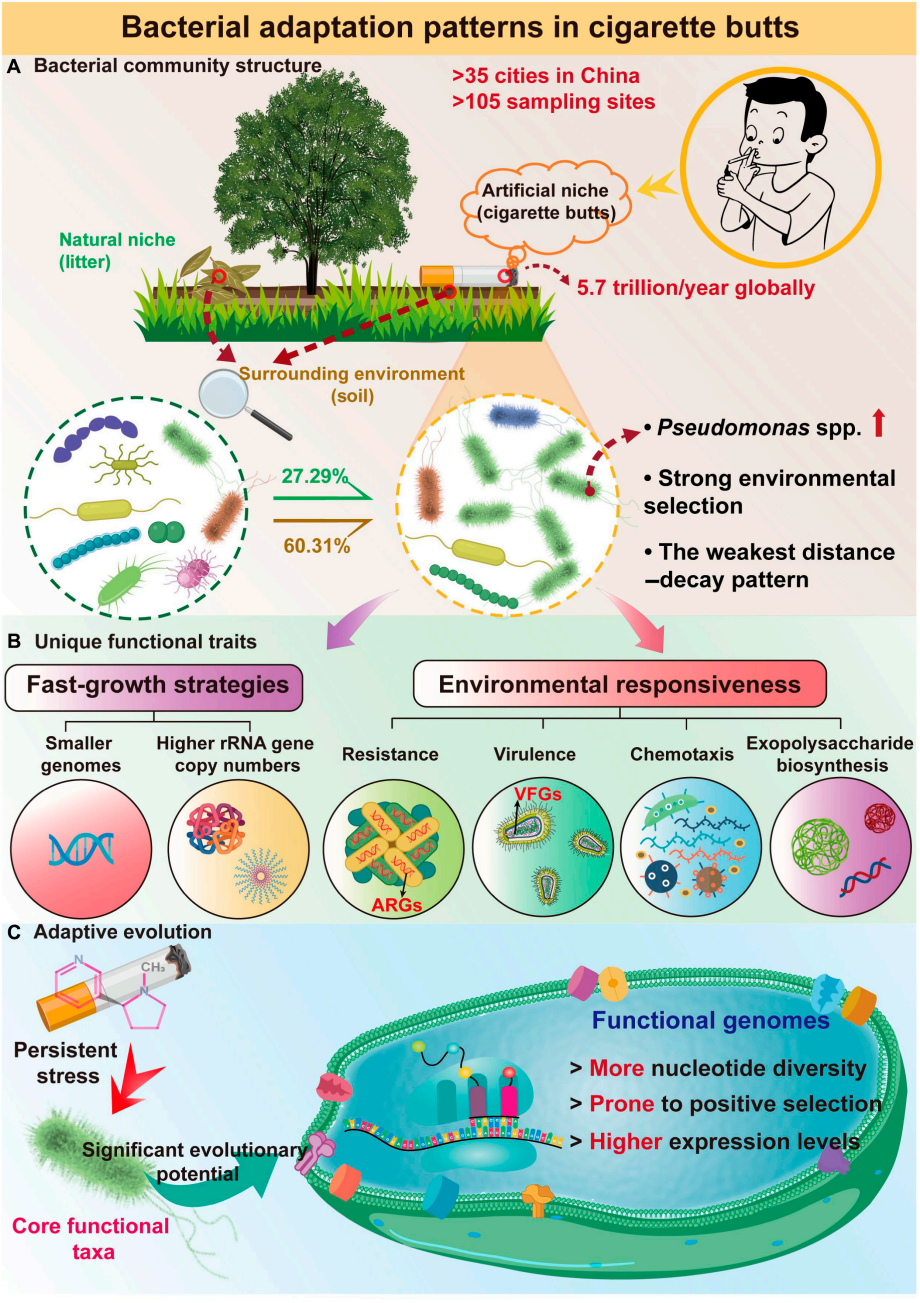
Conceptual diagram of microbial adaptation patterns in cigarette butts. (A) Microbial community structure in the litter (natural niche) and surrounding soil versus cigarette butts (artificial niche), showing strong environmental selection, increased abundance of *Pseudomonas* spp., and the weakest distance–decay pattern. (B) Unique functional traits of cigarette butt-associated microbes, i.e., fast-growth strategies (e.g., smaller genomes and higher ribosomal RNA [rRNA] gene copy numbers) and environmental responsiveness traits (e.g., antibiotic resistance, virulence, chemotaxis, and exopolysaccharide biosynthesis), including enriched antibiotic resistance genes (ARGs) and virulence factor genes (VFGs). (C) Persistent pollution stress (exposure to cigarette butts) drives adaptive evolution in core species (Pseudomonadaceae), increasing nucleotide diversity, positive selection, and expression of functional genomes.

Our study revealed that the bacterial communities within cigarette butts (artificial niche) differed in terms of composition from those in the litter (natural niche) and surrounding soil and were strongly influenced by environmental selection, displaying unique adaptive strategies. The bacterial communities in cigarette butts showed a significantly higher abundance of Proteobacteria (family Pseudomonadaceae in particular) compared with the communities in natural niche. Pseudomonadaceae members, particularly those belonging to the genus *Pseudomonas*, are known for their strong competitive ability, pollutant-degrading capabilities, and environmental stress tolerance [34,35]. Their dominance suggests that the cigarette butt environment imposes strong selective pressure, favoring microorganisms with superior competitive strategies and adaptive traits. Although source tracking analysis indicated that most bacteria in cigarette butts originated from the soil, PCoA results revealed substantial community restructuring within cigarette butts. Further analysis of the microecological mechanisms involved in bacterial community assembly revealed that the communities in cigarette butts exhibited the weakest geographic DDR and the lowest fit to the NCM (Figs. 1 and 6A). This suggests that bacterial community assembly in cigarette butts is primarily driven by strong deterministic environmental selection pressures [36], which may be associated with cigarette butt-specific microenvironmental conditions, including the potential release of compounds such as nicotine and heavy metals, as reported in previous studies [3]. This pattern contrasts with previous plastisphere studies showing that stochastic processes, dispersal limitation, or spatial isolation dominate microbial assembly on polyethylene-film and marine microplastic surfaces [37,38]. Although plastisphere systems can form “bacterial island” effects with enriched specific taxa and functional potentials [37], cigarette butts in our study showed stronger deterministic filtering, highlighting their distinct ecological role in urban environments.

Functional trait analyses further indicated that the bacterial communities in cigarette butts had unique life-history strategies, and their genomic and functional characteristics were significantly different from those observed in the communities of natural niche. Specifically, they exhibited features such as smaller genome sizes and higher rRNA gene copy numbers (Fig. S5), which are often associated with rapid proliferation and environmental responsiveness [39,40]. Further analyses of high-quality MAGs revealed that the bacterial communities from cigarette butts exhibited both faster growth rates and stronger codon usage bias (Fig. 3A and B); key traits that collectively indicate a typical fast-growing ecological strategy [41]. Fast-growing strategies enable microorganisms to rapidly respond to pollution stress [27,42]. This cigarette butt-specific microenvironment may favor stress-tolerant and fast-growing species, thereby driving adaptive evolutionary patterns distinct from those observed in the litter environment.

We found that the bacterial communities in cigarette butts were significantly enriched in functional genes related to antibiotic resistance, pathogenicity, chemotaxis, motility, EPS biosynthesis as well as bacterial biomass and oligosaccharide degradation. Such enrichment suggests a high degree of environmental adaptability, as it confers key ecological advantages, such as stress resistance, rapid resource acquisition, spatial mobility, and enhanced bacterial competition, all of which are crucial for survival and persistence under stressful microenvironmental conditions associated with cigarette butts [25]. These functional traits may enable microbes to efficiently acquire resources, maintain ecological niches, and enhance their competitive advantages within cigarette butt-associated microhabitats. As a persistently disturbed anthropogenic niche, cigarette butts may also be characterized by unstable resource availability and microenvironmental fluctuations, which could impose ecological selection pressures on bacterial communities inhabiting these microhabitats. Under these conditions, microbes must possess traits such as rapid reproduction, effective dispersal, and strong environmental tolerance and competitiveness (e.g*.*, chemotaxis, motility, antibiotic resistance, and EPS secretion) [43] to achieve rapid colonization and niche establishment. This means that they must exhibit a higher number of genomic features associated with fast growth (R-strategy) and competition-stress tolerance (CS-strategy) [24,26] (Fig. 6B). These life-history strategies reflect a coordinated response involving opportunistic invaders and pollution-adapted taxa, in which resistance and pathogenicity provide key competitive advantages for survival in the cigarette butt environment [43]. In line with this, functional genes associated with these traits were found to be significantly enriched in the genomes of cigarette butt-associated bacteria, especially within dominant members of phylum Proteobacteria (e.g., Pseudomonadaceae), a lineage widely recognized for stress tolerance and competitive ability [44]. Collectively, these features indicate that the cigarette butt microenvironment may impose ecological selective pressure, favoring competitive life-history strategies characterized by resistance and pathogenic potential, thereby driving community adaptation in this anthropogenic microenvironment. In contrast, the litter microbiome typically harbors larger genomes and fungal biomass-degrading enzymes, which reflect a strategy focused on metabolism and resource acquisition [25]. These findings support our first scientific hypothesis: Urban cigarette butts constitute a distinct ecological niche that is fundamentally different from natural litter environments and that reshapes the unique life-history strategies of bacterial communities within it.

Our results further showed that, compared with the soil and litter niches, the genomes of bacterial communities associated with cigarette butts exhibited higher nucleotide diversity, a greater number of SNVs, and a higher proportion of genes under positive selection, indicating a more intense genomic variation and selective pressure [45]. Notably, these differing genetic features mainly affected key functional pathways related to antibiotic resistance, human pathogenicity, chemotaxis, EPS biosynthesis, and bacterial biomass degradation. Further analysis revealed that members of the phylum Proteobacteria, especially the family Pseudomonadaceae, demonstrated significant evolutionary potential in these functional categories, being characterized by elevated nucleotide diversity, higher pN/pS ratios, and increased SNV accumulation. Pseudomonadaceae also showed a markedly higher proportion of positively selected genes regulating antibiotic resistance, motility, and pathogenicity pathways compared with other bacterial families, along with greater microdiversity in genes regulating chemotaxis and EPS biosynthesis. This potential was further supported by the controlled pure culture experiment. Compared with the natural substrate and inert artificial surface controls, *P. aeruginosa* exposed to cigarette butts showed significant up-regulation in the expression of key functional genes, including those related to motility, biofilm formation, virulence factors, and antibiotic resistance. These findings further suggest that cigarette butts may impose ecological selective pressures on bacterial communities and induce adaptive functional responses in *Pseudomonas*. Thus, the family Pseudomonadaceae may represent an important bacterial lineage with adaptive potential in cigarette butt-associated microhabitats (Fig. 6C). Collectively, these results provide strong support for our second scientific hypothesis that the cigarette butt environment promotes adaptive evolution in core bacterial taxa, particularly in genes associated with essential ecological functions. This evolutionary pattern resembles bacterial adaptation in extreme environments such as saline-alkaline soils, contaminated farmlands, and rhizospheres at mining sites [46]. In saline soils, halophilic bacteria such as genera *Halomonas* and *Marinobacter* reduce energy expenditure through genome streamlining and osmotic regulation, whereas halophilic archaea enhance carbon acquisition to expand niche breadth [47]. In polluted soils and within rhizospheres at mining sites, tolerant taxa such as Proteobacteria and Chloroflexi have a high number of resistance genes, exhibit enhanced chemotaxis, and maintain carbon and nitrogen metabolism to survive under nutrient limitation and chemical stress [48,49]. In the cigarette butt environment where nutrients are scarce, bacterial communities are driven toward rapid evolution and high tolerance, exhibiting genomic variation and adaptive traits similar to those observed in communities living in those extreme natural habitats.

Our study highlights the unique ecological and evolutionary roles of cigarette butts as an anthropogenic microhabitat within urban microbial ecosystems. The microbial communities within cigarette butts were found to be compositionally distinct from those in the litter (a natural niche) and characterized by rapid growth, strong competitiveness, and high stress tolerance. Cigarette butt-associated microhabitats may be linked to adaptive evolutionary patterns in core bacterial taxa, particularly members of Pseudomonadaceae. These findings reveal that cigarette butts act as overlooked yet potent selective environments, favoring stress-tolerant, competitive, and potentially pathogenic microbes and thereby posing challenges to the health of urban microbial ecosystems. By integrating ecological trait analyses with evolutionary indicators, this study provides a comprehensive framework for understanding how anthropogenic pollutants reshape microbial functional turnover and evolutionary trajectories in urban ecosystems.

A limitation of this study is that the chemical properties of cigarette butts were not directly measured. Therefore, the observed microbial community shifts, functional enrichment, and genomic microdiversity patterns should be interpreted as responses to cigarette butt-associated microhabitats rather than as direct evidence of selection by specific chemical contaminants. In addition, our controlled pure culture experiment included wooden sticks and glass rods as natural substrate and inert artificial surface controls, respectively, providing supporting evidence that cigarette butt exposure induced functional responses beyond general surface colonization or natural substrate effects. Nevertheless, additional controls, such as unused cigarette filters and pure cellulose acetate materials, would further help distinguish the effects of cigarette butt-specific chemicals from those of filter material and general surface properties. Moreover, because this experiment was conducted using a single model strain, *P. aeruginosa* PAO1, under laboratory conditions, it provides supporting evidence that cigarette butt exposure may induce functional responses in *Pseudomonas* but should not be interpreted as direct proof of community-level niche differentiation in field-associated Pseudomonadaceae communities. Future studies integrating chemical characterization, unused filter and cellulose acetate controls, multiple environmental isolates, and in situ experimental systems are needed to identify the specific drivers of these microbial responses.

## Materials and Methods

### Sites and sample collection

Samples of cigarette butts, surrounding aged plant litter (hereafter referred to as the “litter”), and surface soil were collected from 105 urban green spaces across 35 cities in China during sunny days in October and November 2022. In each city, 3 urban parks were randomly selected as sampling sites (Fig. 1A). Within each park, 3 1 m × 1 m quadrats were randomly established in vegetated areas where cigarette butts were visibly present, typically near pedestrian paths, benches, or trash bins. To investigate the life-history traits of microorganisms colonizing cigarette butts and distinguish these microbes from those present in natural niches such as the litter, only visibly aged cigarette butts were targeted. These were defined as cellulose acetate filter-tipped butts that showed clear signs of environmental exposure and weathering (e.g., discoloration, partial fraying of fibers, or light surface soiling) but lacked extensive physical disintegration or overgrowth by macroorganisms. This careful selection ensured that the samples harbored microbial communities adapted to prolonged exposure to the cigarette butt environment while maintaining structural integrity for downstream processing.

In each quadrat, 2 to 3 cigarette butts and the litter adjacent to them were collected, for a total of approximately 6 to 9 cigarette butts sampled per park. Surface soil samples (0- to 10-cm depth) were also collected using a soil auger with a diameter of 5 cm. The soil from each quadrat was homogenized to form a composite sample. All samples were placed in sterile sampling bags, transported in insulated boxes with ice packs under chilled conditions, and immediately delivered to the laboratory for further processing upon completion of sampling in each city. In the laboratory, soil samples were sieved through a 2-mm mesh after removing visible plant debris and stones. One portion was air-dried at room temperature for physicochemical analyses, including pH measurement and dissolved organic carbon quantification using a Mettler Toledo pH5 meter (Switzerland) and a total organic carbon analyzer (Multi N/C 3100, Analytik Jena, Germany), respectively.

### DNA extraction and full-length 16S rRNA gene sequencing

Total DNA was extracted from the cigarette butt, litter, and soil samples using the FastDNA Spin Kit for soil (MP Biomedicals, USA) following the manufacturer’s protocol [50]. The cigarette butt samples were specifically pretreated as previously described [35,51]. In brief, the filters were carefully cut out from the surrounding paper using sterilized scissors, and any remaining tobacco particles were removed with the aid of sterile tweezers and pressurized air. Six pretreated cigarette butts collected from each park were placed into a sterile 250-ml Erlenmeyer flask containing 100 ml of 0.01 M sterile phosphate-buffered saline and subjected to shaking at 180 rpm for 2 h at 25 °C. The resulting suspension was sequentially filtered through a sterile nylon mesh and a 0.22-μm cellulose membrane. The filter membranes were cut into small pieces using sterilized scissors, and DNA was extracted from the filter-colonizing microbial communities using the abovementioned kit.

Similarly, for the litter samples [35], 5 g of leaf material was placed in a 250-ml Erlenmeyer flask with 100 ml of 0.01 M phosphate-buffered saline. The sample was shaken to release surface-associated microorganisms, and the suspension was filtered first through a sterile nylon net and then through a 0.22-μm cellulose membrane. The filter membranes thus obtained were cut into fragments, and DNA was extracted using the same kit mentioned above. All DNA extracts were stored at −80 °C until further processing. All samples collected from 105 urban parks across multiple cities were subjected to full-length 16S rRNA gene amplification and sequencing. In addition, another set of samples (cigarette butts, litter, and soil) was obtained from 1 representative park randomly selected in each city (35 parks in total) and subjected to metagenomic sequencing, resulting in a total of 105 metagenomic datasets.

Full-length 16S rRNA genes were amplified using the universal bacterial primers 27F (5′-AGAGTTTGATCMTGGCTCAG-3′) and 1492R (5′-GGTTACCTTGTTACGACTT-3′) [52], each tagged with PacBio barcodes. polymerase chain reaction products were purified by electrophoresis, pooled at equimolar concentrations, and used to construct SMRTbell libraries (Prep Kit 3.0, Pacific Biosciences). Sequencing was conducted on the PacBio Sequel IIe platform, and high-accuracy circular consensus reads were generated in SMRT Link v11.0. Amplicon sequence variants were inferred using DADA2_CCS [53], and taxonomy was assigned using the Silva 138 database with a confidence threshold of 0.7.

### Metagenomic contig-based analysis of bacterial life-history strategies

Paired-end metagenomic libraries were constructed from total DNA using the Tecan Ovation Ultralow System V2 [54] and were sent to Majorbio Bio-Pharm Technology Co., Ltd. (Shanghai, China) for sequencing on the Illumina NovaSeq 6000 platform [55]. Raw reads were quality-filtered using Fastp [54] and assembled into contigs in MEGAHIT v1.2.9 [56,57]. Contigs of potential eukaryotic origin were removed using EukRep v0.6.7 [58], and the remaining prokaryotic contigs were functionally annotated against the Kyoto Encyclopedia of Genes and Genomes (KEGG) database alongside the Non-supervised Orthologous Groups (eggNOG), and CAZy databases (DIAMOND, e-value ≤ 1e−5) [54] to identify genes associated with metabolism and ecological functions, as detailed in Data S2. Gene abundances were further calculated by mapping clean reads to predicted genes using BBMap [55]. Antibiotic resistance genes and virulence factor genes were identified by aligning clean reads to the Structured Antibiotic Resistance Gene (SARG) [59] and Virulence Factors of Pathogenic Bacteria (VFDB) [60] databases using DIAMOND BLASTX [61], and their abundances were normalized as copies per cell.

To investigate the life-history traits of bacteria inhabiting cigarette butts, the litter, and soil, we drew on established frameworks for exploring microbial life-history strategies [24,26,62]. Based on these frameworks, we constructed a bacterial life-history trait matrix by integrating functional categories from multiple databases, including KEGG, CAZy, eggNOG, SARG, and VFDB. Trait categories included basic metabolism, chemotaxis, sigma factors, biofilm formation and adhesion, siderophore biosynthesis, molecular chaperones, motility, antibiotic resistance, pathogenic interactions with human, carbohydrate degradation, quorum sensing, nutrient uptake systems, membrane biosynthesis, and sporulation. Detailed classifications are provided in Data S3 to S5. In addition, we derived genome-level ecological strategy indicators from contigs, including estimated average genome size, GC content, and 16S rRNA gene copy number. Average genome size was estimated using the Microbe Census pipeline [63], GC content was calculated using BBTools [27] from G + C base ratios, and 16S rRNA copy number was determined via Barrnap [64,65].

### Metagenomic binning, microdiversity profiling, and genomic identity index estimation

Assembly quality was evaluated via QUAST [30], and contigs were binned into MAGs using the binning module in MetaWRAP [66]. MAG quality was assessed using CheckM [67], and only high-quality MAGs (≥90% completeness and ≤5% contamination) were retained for subsequent analyses. These MAGs were dereplicated at 95% [68] average nucleotide identity using dRep [55] and taxonomically annotated via GTDB-Tk (classify_wf workflow) [55]. A maximum likelihood phylogenetic tree was generated using IQ-TREE based on bacterial marker genes identified via GTDB-Tk [69]. Clean reads were mapped to MAGs using CoverM [70] to estimate relative abundance, expressed as reads per kilobase per million mapped reads [55]. Protein-coding sequences within MAGs were identified using Prodigal v2.6.3 [71] and compared against the KEGG, CAZy, eggNOG, SARG, and VFDB databases via BLASTX in DIAMOND v2.0.14.152 [61], applying thresholds for identity ≥ 60%, coverage ≥ 70%, and e-value ≤ 10^−5^. The maximal growth rate and codon usage bias of high-quality MAGs were estimated using gRodon [54], and genome size was assessed via CheckM [68].

Microdiversity metrics, including genome-wide and gene-level nucleotide diversity, SNVs, and the ratio of nonsynonymous to synonymous mutations (pN/pS), were calculated using InStrain v1.0.0 [72], with high-quality MAGs used as the reference database.

### Adaptive mechanisms of *P. aeruginosa* under exposure to cigarette butts: Laboratory analyses

To avoid the involvement of human smokers and minimize interindividual variability, smoked cigarette butts were generated using a smoking simulator following the method described in previous studies [73,74]. Specifically, Zhonghua cigarettes, a widely consumed brand produced in China, were selected as the experimental material. Each cigarette was secured to a 20-ml syringe with adhesive putty. The syringe plunger was then repeatedly pulled and pushed to mimic smoking until the cigarette burned to approximately 1 cm from the filter. The resulting butts were collected using sterile forceps, and the surrounding paper was carefully removed using sterile scissors. Then, residual ash was cleared with the aid of sterile tweezers and pressurized air. The collected cigarette butts were sterilized by autoclaving prior to experiments. Once sterilized, they were used along with size-matched sterilized glass rods and wooden sticks as substrates for biofilm formation.

*P. aeruginosa* PAO1 was selected because Pseudomonadaceae, particularly *Pseudomonas*, was enriched in cigarette butt-associated microbial communities in our field samples. Moreover, *P. aeruginosa* is a well-established model organism for studying gene expression, quorum sensing, antibiotic resistance, virulence, and biofilm formation, making PAO1 suitable for evaluating adaptive functional responses to cigarette butt exposure [75]. A single colony of *P. aeruginosa* PAO1 was inoculated into 10 ml of Luria–Bertani broth and cultured at 37 °C for 12 h to reach a cell density of approximately 10^8^ to 10^9^ CFU/ml. Then, 1 ml of this culture was diluted into 300 ml of fresh Luria–Bertani medium to achieve an initial concentration of ~10^6^ CFU/ml. One unit of each substrate type was aseptically transferred into separate 500-ml Erlenmeyer flasks containing the diluted culture, sealed with sterile parafilm, and labeled as PC (cigarette butt group), PG (glass control), and PW (wood control). All flasks were incubated at 35 °C with shaking at 200 rpm for 30 d to allow biofilm development. At the end of incubation, biofilm samples were collected from the material surfaces, transferred into sterile 5-ml cryotubes, and immediately flash-frozen in liquid nitrogen for subsequent RNA extraction and metatranscriptomic sequencing. RNA was extracted from the biofilm samples using the RNeasy Mini Kit (Qiagen, Germany) and sequenced on the NovaSeq 6000 platform to generate 150-bp paired-end reads [54]. Low-quality and short reads were filtered in Trimmomatic (v0.36) [76], and rRNA reads were removed using SortMeRNA against the SILVA Small Subunit and Large Subunit ribosomal RNA databases [77]. The remaining reads were assembled de novo in Trinity (v2.4.0) [78], clustered via Cluster Database at High Identity with Tolerance to obtain a unigene catalog [79], and mapped back using Bowtie2 to quantify gene expression [80]. Functional annotation was performed against the KEGG, eggNOG, and CAZy databases, and differential expression was analyzed via DESeq [81]. Each group included 3 biological replicates.

### Statistical analysis

MCOA was performed using the R package ade4 [25,82] to identify the major axes underlying global variation in the community-aggregated traits of soil bacteria [83]. This method integrates trait information from 6 databases (life-history traits, eggNOG, KEGG, CAZy, SARG, and VFDB) by maximizing the shared structure across multiple multivariate tables. All trait variables were standardized using *z*-score transformation prior to analysis. In the MCOA, the first 2 axes were extracted and used as latent variables representing bacterial community positions in the trait dimensions. Random forest models were then built to identify key environmental drivers of these trait dimensions [84], including soil physicochemical properties, geographic coordinates, and microbial community composition. Microbial alpha diversity was assessed using the “vegan” package (v4.0.3) [66] in R. Differences in community composition among the cigarette butt, litter, and soil samples were evaluated using ANOSIM. The microbial source-tracking method fast expectation-maximization microbial source tracking was applied to trace the potential sources of microbiota in the samples. PCoA based on Bray–Curtis dissimilarity was conducted to visualize patterns in microbial community structure across the 3 niches examined. Data were visualized using Chiplot (https://www.chiplot.online/), Origin 2024b, and R (v4.0.3).

## Data Availability

Metagenomic reads, 16S rRNA gene, and metatranscriptomics sequencing reads generated in this study were deposited in the National Center for Biotechnology Information, with accession numbers PRJNA1072117, PRJNA1073257, and PRJNA1310479, respectively.

## Supplementary Materials

Supplementary 1 Graphical Abstract Figs. S1 to S13 Data S1 to S5
