# Supplementary material for: Cigarette Butts as an Emerging Urban Habitat Driving Microbial Niche Differentiation
Source: Research (Wash D C). 2026 Jul 27;9:1380. doi: 10.34133/research.1380 (PMC13402723; doi:10.34133/research.1380)
Supplement: Supplementary 1 — Graphical Abstract Figs. S1 to S13 Data S1 to S5 [file research.1380.f1.zip › Supplementary Material.doc.docx]

**Supporting Information for**

Cigarette butts as an emerging urban habitat driving microbial niche differentiation

Ting Xie^1,2^, Jia-Yang Xu^1,2^, Da Lin^1,3*^, Yang Liu^4^, Yi-Fei Wang^1,2^, Zhu-Gen Yang^5^, Patrick K. H. Lee^6^, Dong Zhu^1,2*^

^1^State Key Laboratory of Regional and Urban Ecology, Ningbo Urban Environment Observation and Research Station, Institute of Urban Environment, Chinese Academy of Sciences, Xiamen 361021, China.

^2^Zhejiang Key Laboratory of Pollution Control for Port-Petrochemical Industry, CAS Haixi Industrial Technology Innovation Center in Beilun, Ningbo 315830, China.

^3^University of Chinese Academy of Sciences, Beijing 100049, China.

^4^School of Marine Sciences, Ningbo University, Ningbo 315211, China.

^5^Faculty of Engineering and Applied Sciences, Cranfield University, Milton Keynes MK43 0AL, U.K.

^6^School of Energy and Environment and State Key Laboratory of Marine Pollution, City University of Hong Kong, Hong Kong SAR, China.

***Corresponding authors:** Da Lin; Dong Zhu;

**Email**: [dali@iue.ac.cn](mailto:dali@iue.ac.cn); dzhu@iue.ac.cn;

**This PDF file includes:**

Fig. S1 to S13

**Other supporting materials for this manuscript include the following:**

Datasets S1-S5

**Supplementary Figures**


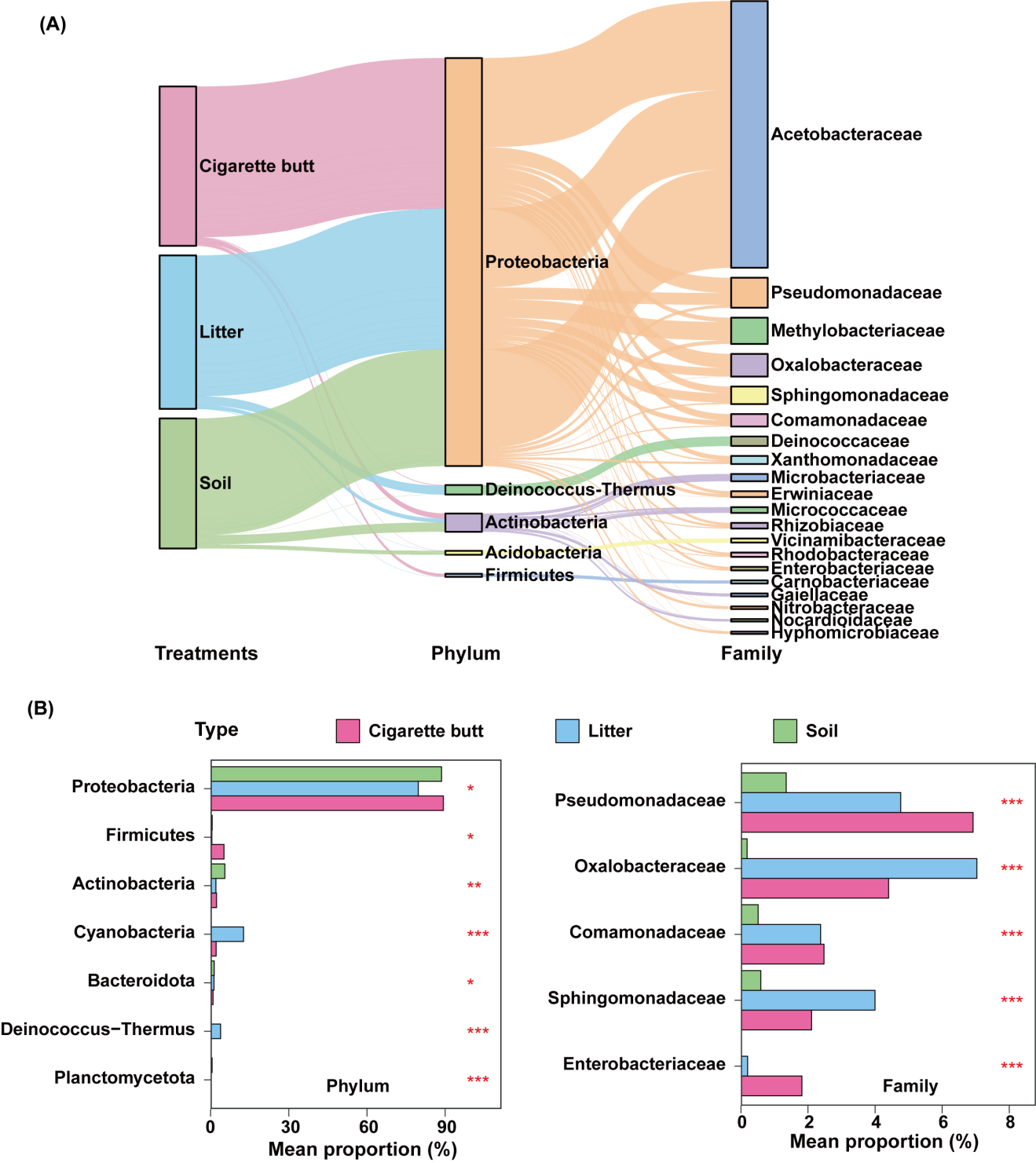


**Fig. S1. Composition of bacterial communities across cigarette butts, litter, and soil samples. (A)** The distribution of bacterial phylum and families across different sample types. Line widths correspond to the relative abundance of each taxon; **(B)** The bacterial phylum and families with significant differences in relative abundance among the sample types (Kruskal–Wallis test, **p* < 0.05, ***p* < 0.01, ****p* < 0.001).


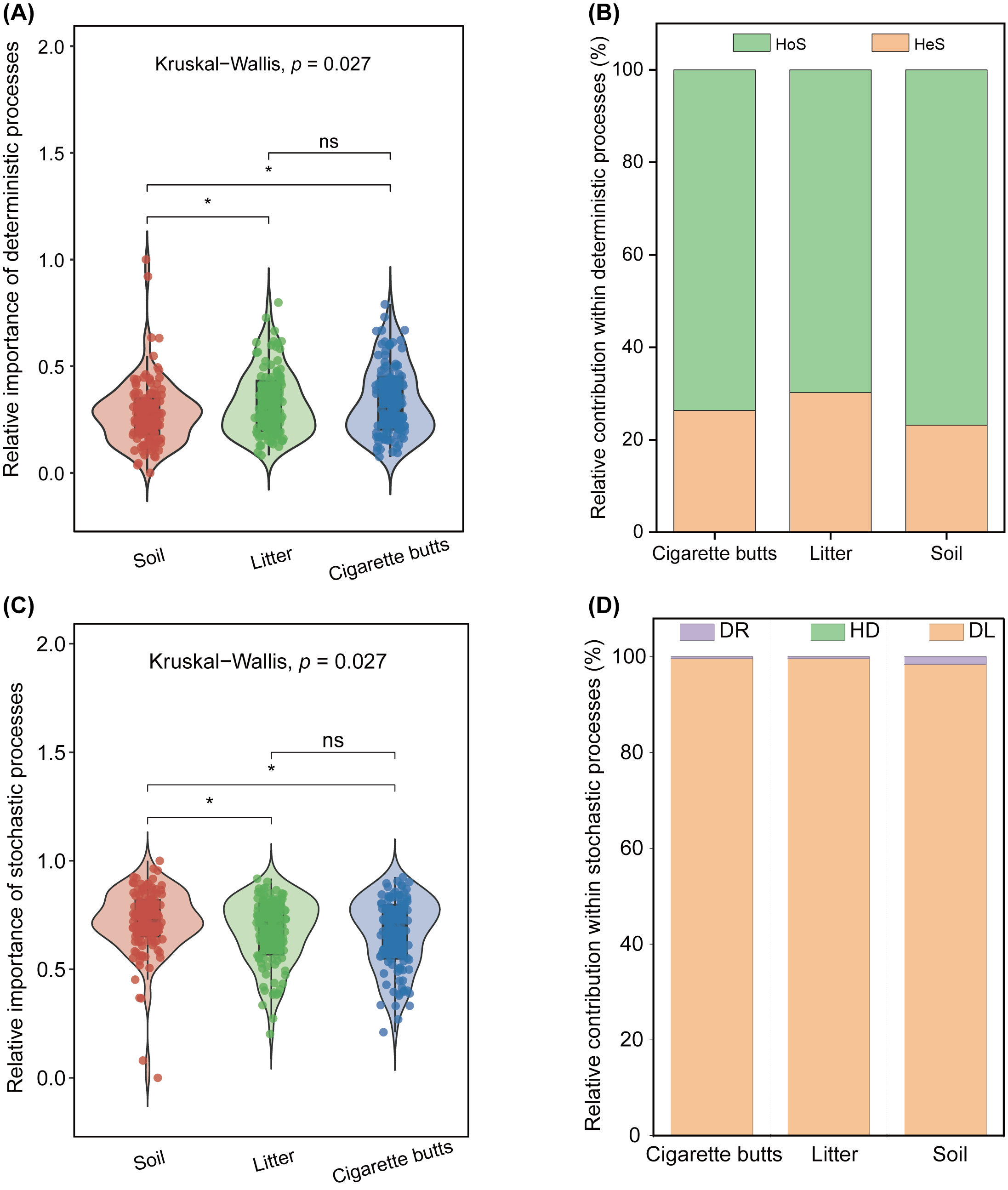


**Fig. S2. iCAMP-based analysis of bacterial community assembly processes across ecological niches.** **(A)** Relative importance of deterministic processes, represented by the sum of heterogeneous selection (HeS) and homogeneous selection (HoS), followed by min–max normalization. **(B)** Relative contributions of HoS and HeS within deterministic processes. **(C)** Relative importance of stochastic processes, represented by the sum of dispersal limitation (DL), homogenizing dispersal (HD), and drift and others (DR), followed by min-max normalization. **(D)** Relative contributions of DL, HD, and DR within stochastic processes.


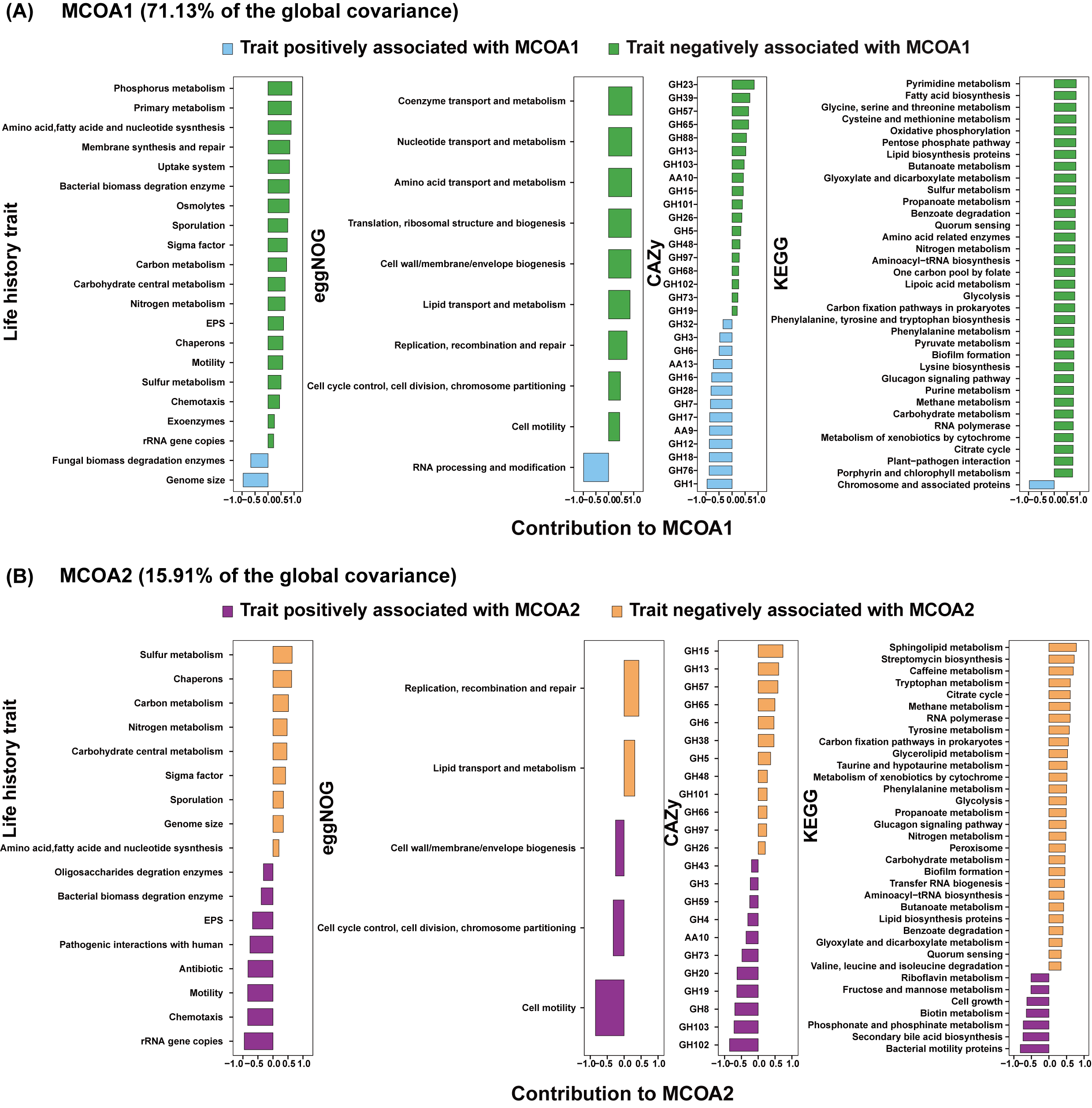


**Fig. S3. Trait dimensions of bacterial metagenomes in cigarette butts, litter, and soil samples. (A)** Variable contributions to MCOA dimensions 1 (A) and 2 (B), summarizing the shared trait structures derived from four CAT databases (Life history trait, eggNOG, CAZy, and KEGG). The top 35 variables are shown for each database when the number of significant variables exceeds 35. Bar plots display the top contributing variables with significant correlations (*p* < 0.05) for each MCOA dimension, highlighting key functional traits within bacterial metagenomes. Bar colours indicate the direction of the associations between each variable and the MCOA dimensions (green/blue for MCOA1; purple/orange for MCOA2).


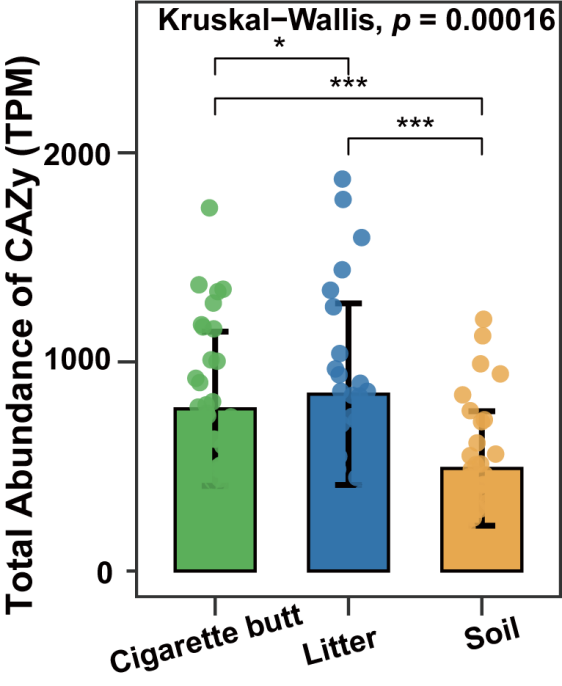


**Fig. S4. Total abundance of CAZy (TPM) in cigarette butts, litter, and soil samples.**


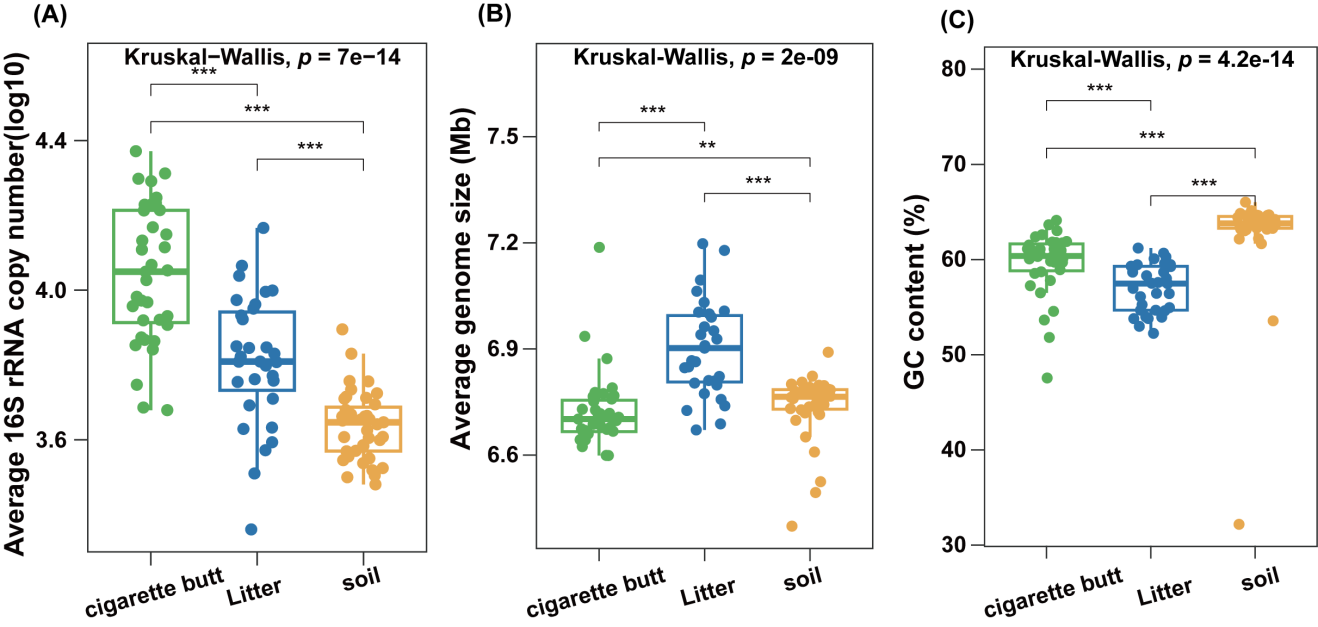


**Fig. S5. Metagenomic traits in cigarette butts, litter, and soil samples. (A)** Average 16S rRNA gene copy number; **(B)** average genome size; and **(C)** GC content. Statistical significance was assessed using the Kruskal–Wallis test (***p* < 0.01; ****p* < 0.001).


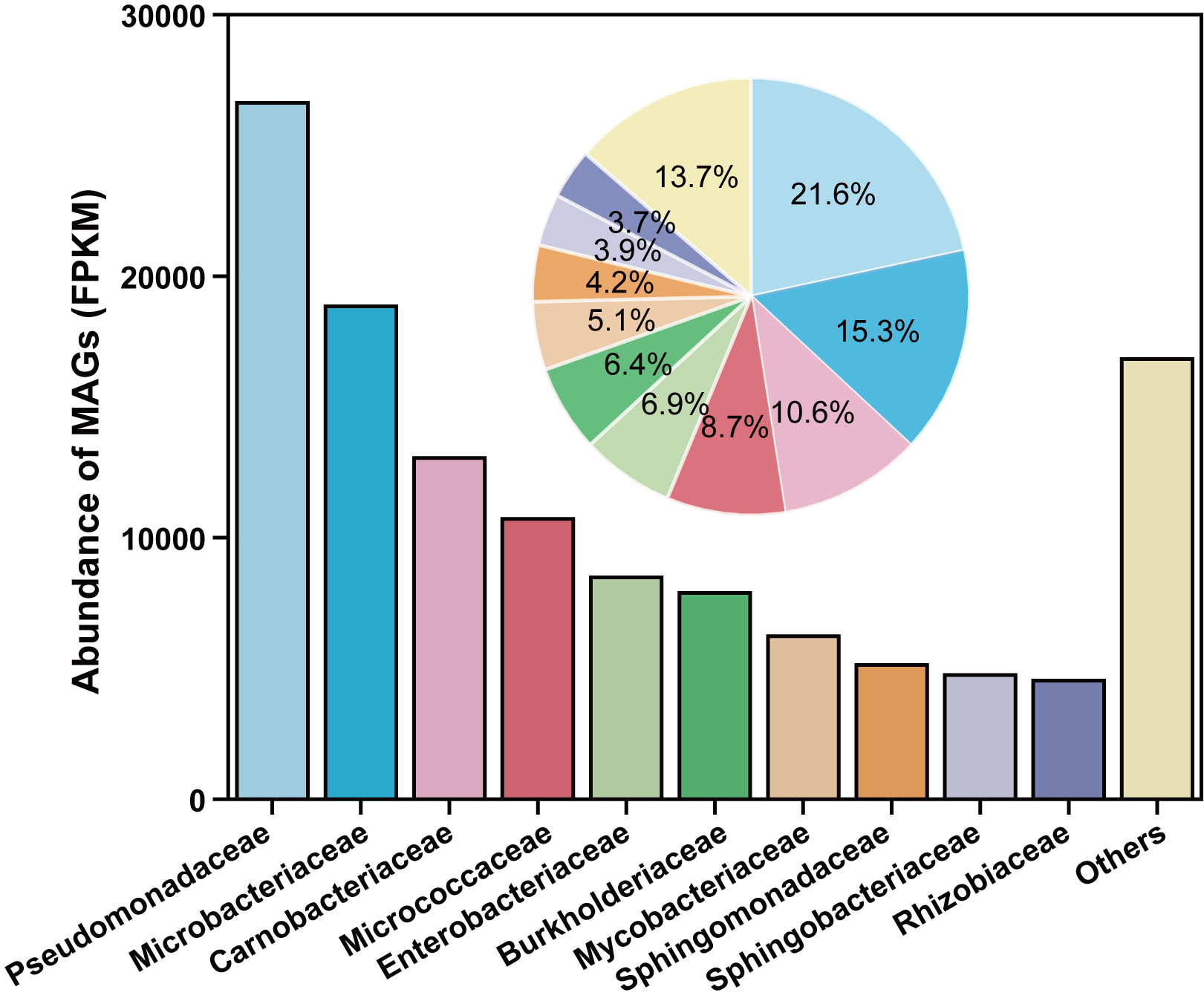


**Fig. S6. Abundance of assembled high-quality bacterial MAGs at the family level in cigarette butts.** The pie chart showed the proportion of total abundance contributed by each species.


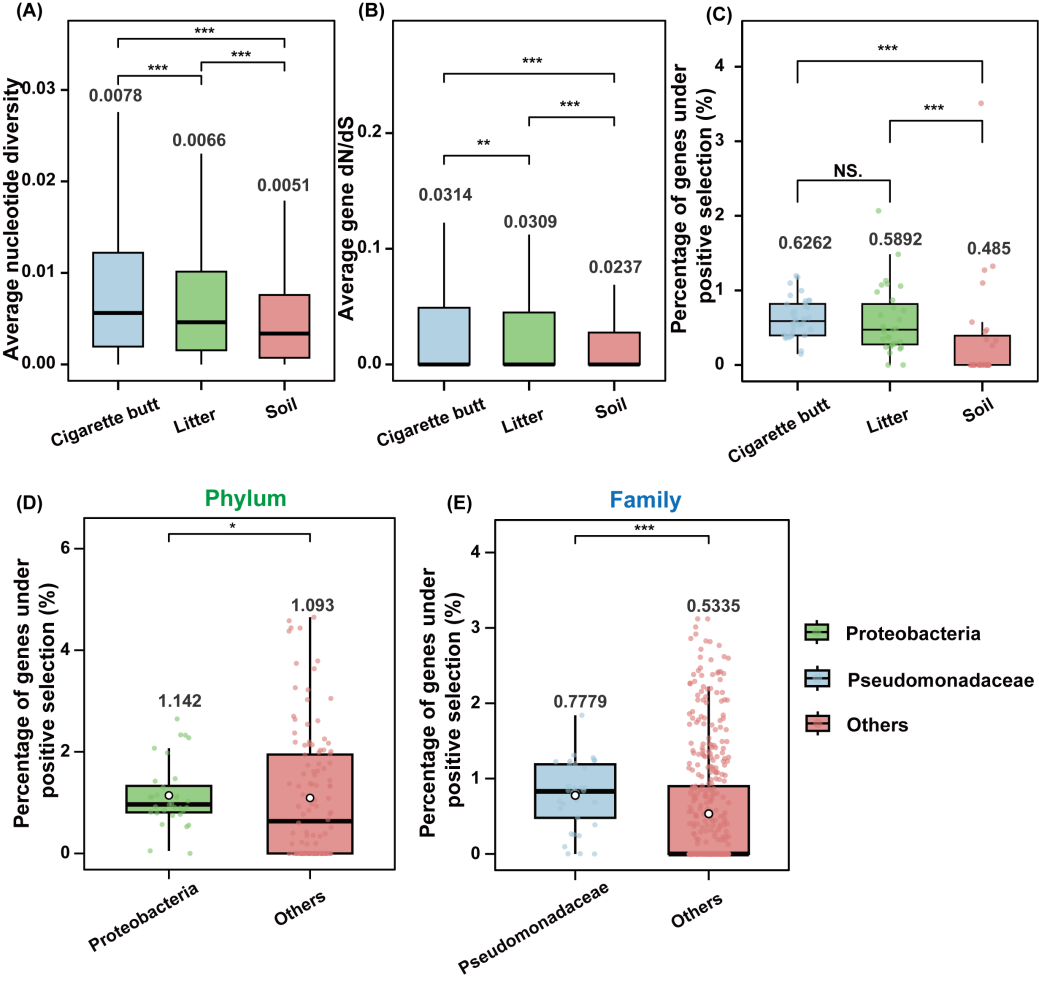


**Fig. S7. Bacterial microdiversity and evolutionary forces across cigarette butts, litter, and soil.** Comparison of average nucleotide diversity **(A)**, average gene dN/dS **(B)**, and percentage of genes under positive selection (pN/pS > 1) of the bacteria genome **(C)** among the three niches; percentage of positively selected genes within Proteobacteria vs. other phylum **(D)**, and within *Pseudomonadaceae* vs. other families **(E)** in bacterial genomes from cigarette butts. Box plots represent the distribution of values across samples; horizontal lines indicate medians, white circles denote means. Statistical differences were assessed using Kruskal–Wallis tests; * *p* < 0.05, ** *p* < 0.01, *** *p* < 0.001; NS, not significant.


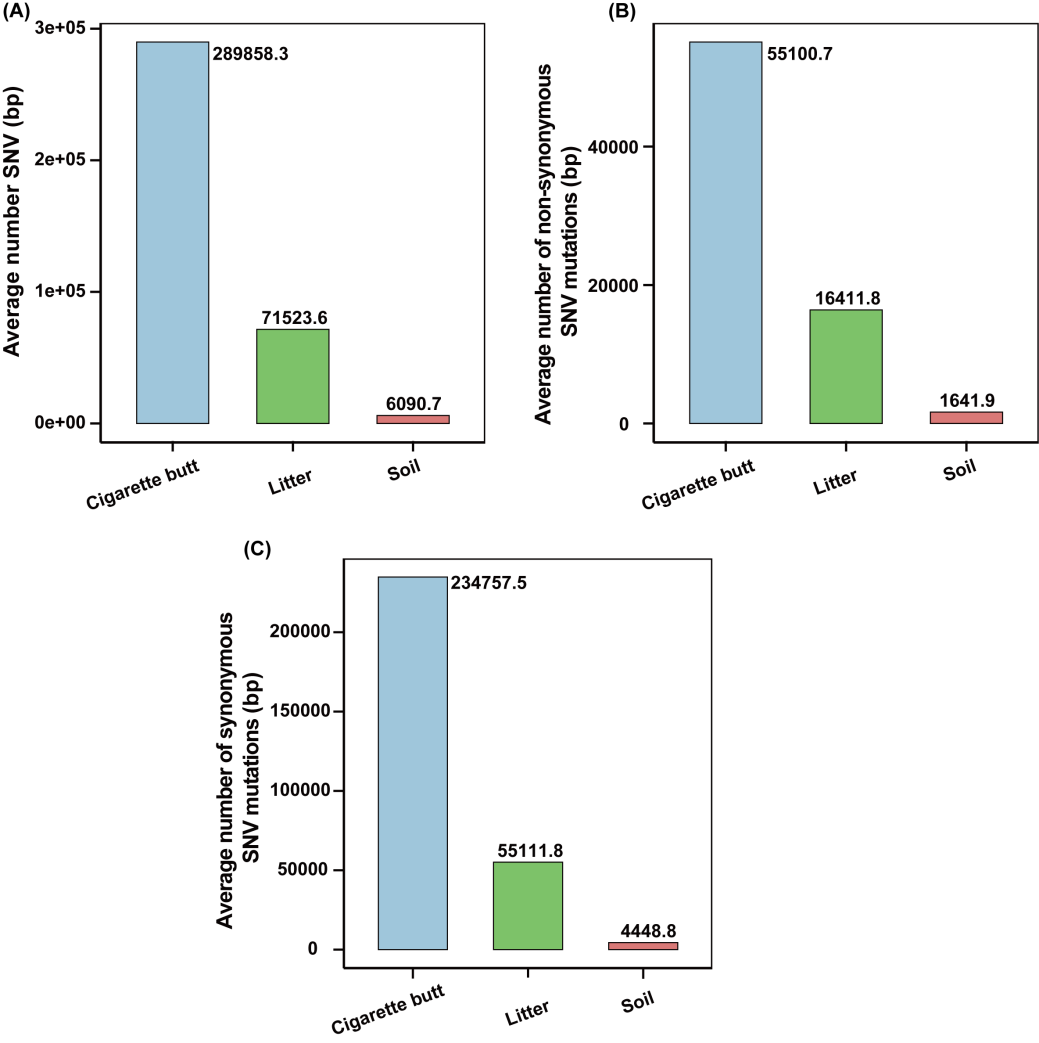


**Fig. S8. Comparison of single nucleotide variant (SNV) mutation types across microbial communities from cigarette butts, litter, and soil. (A)** Average number of total SNVs (bp); **(B)** Average number of non-synonymous SNV mutations (bp); **(C)** Average number of synonymous SNV mutations (bp).


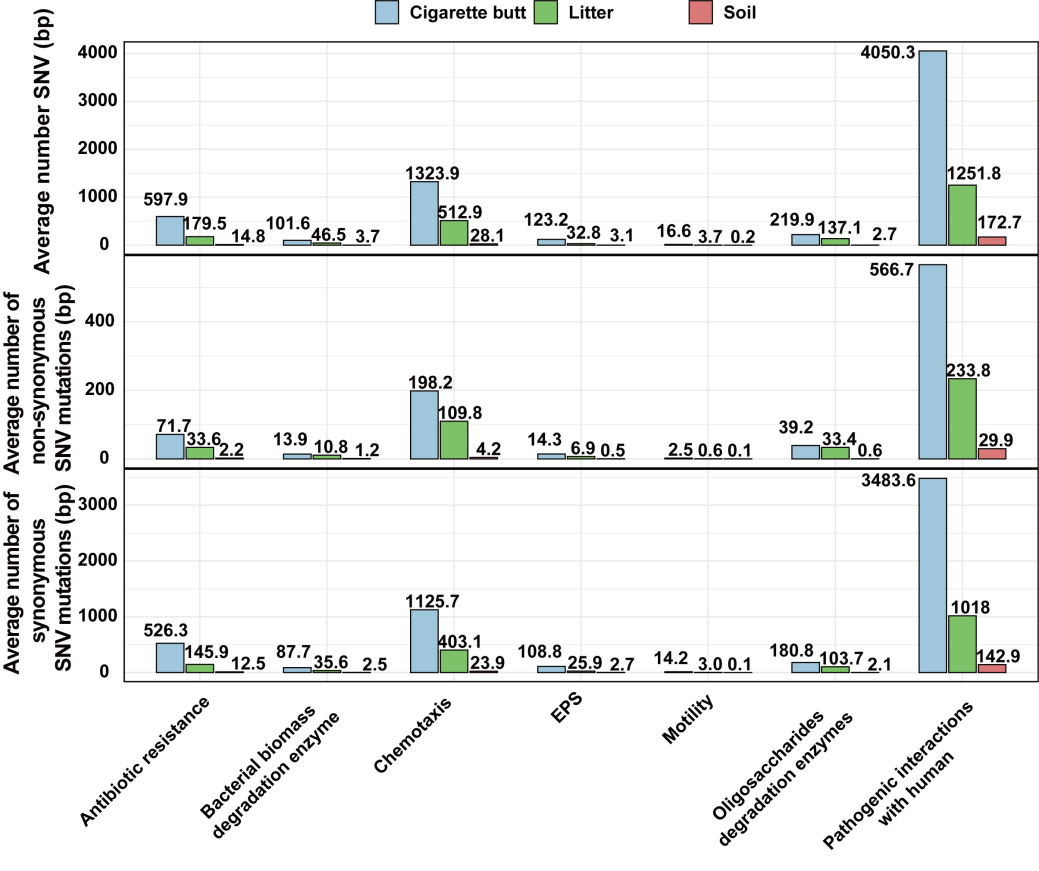


**Fig. S9. Functional category-specific distribution of average SNV, synonymous, and non-synonymous mutation counts across cigarette butts, litter, and soil.**


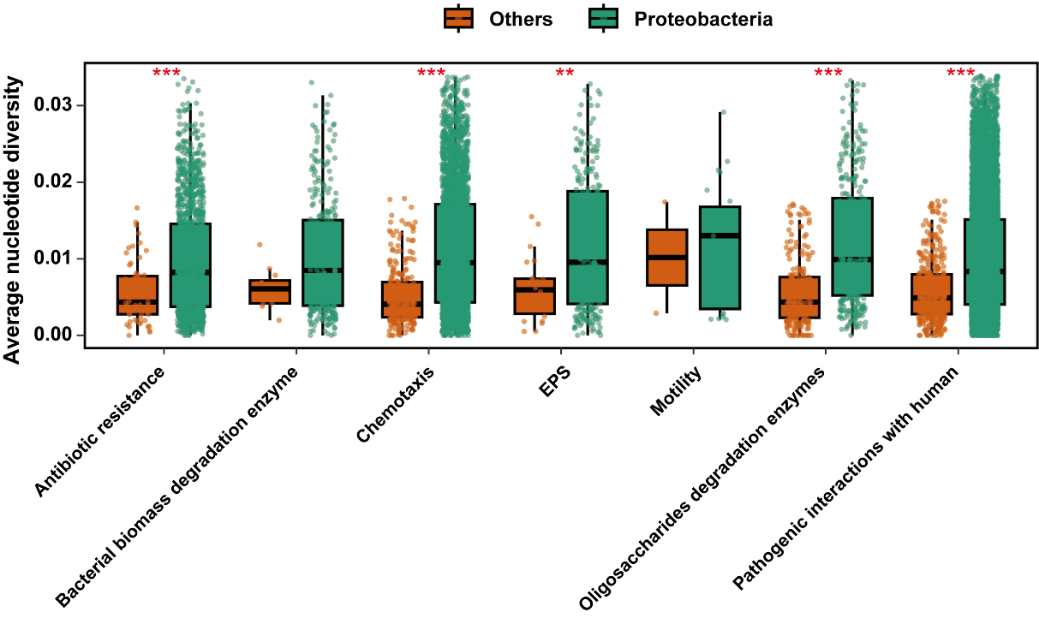


**Fig. S10. Comparison of average nucleotide diversity between Proteobacteria and other bacterial phyla across different functional categories in cigarette butt-associated microbial communities.**


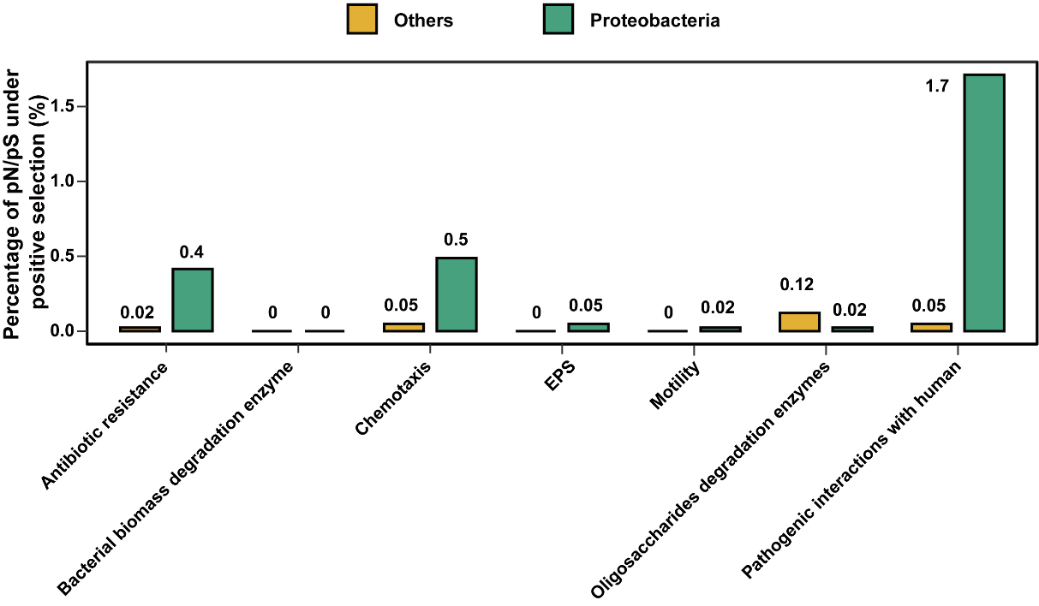


**Fig. S11. Comparison of the proportion of genes under positive selection (pN/pS > 1) between Proteobacteria and other bacterial phylum across various functional categories in cigarette butt-associated microbial communities.**


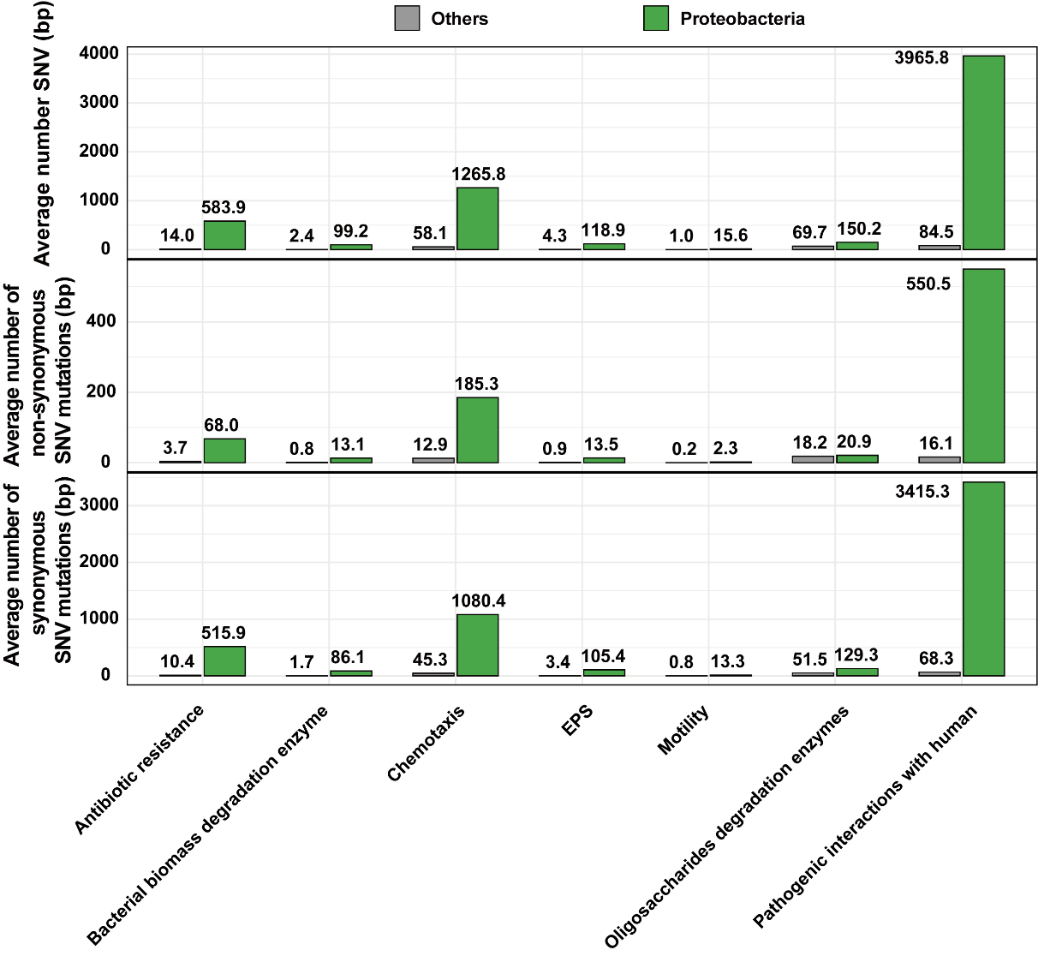


**Fig. S12. Comparison of the average SNV, synonymous, and non-synonymous mutation counts between Proteobacteria and other bacterial phylum across various functional categories in cigarette butt-associated microbial communities.**


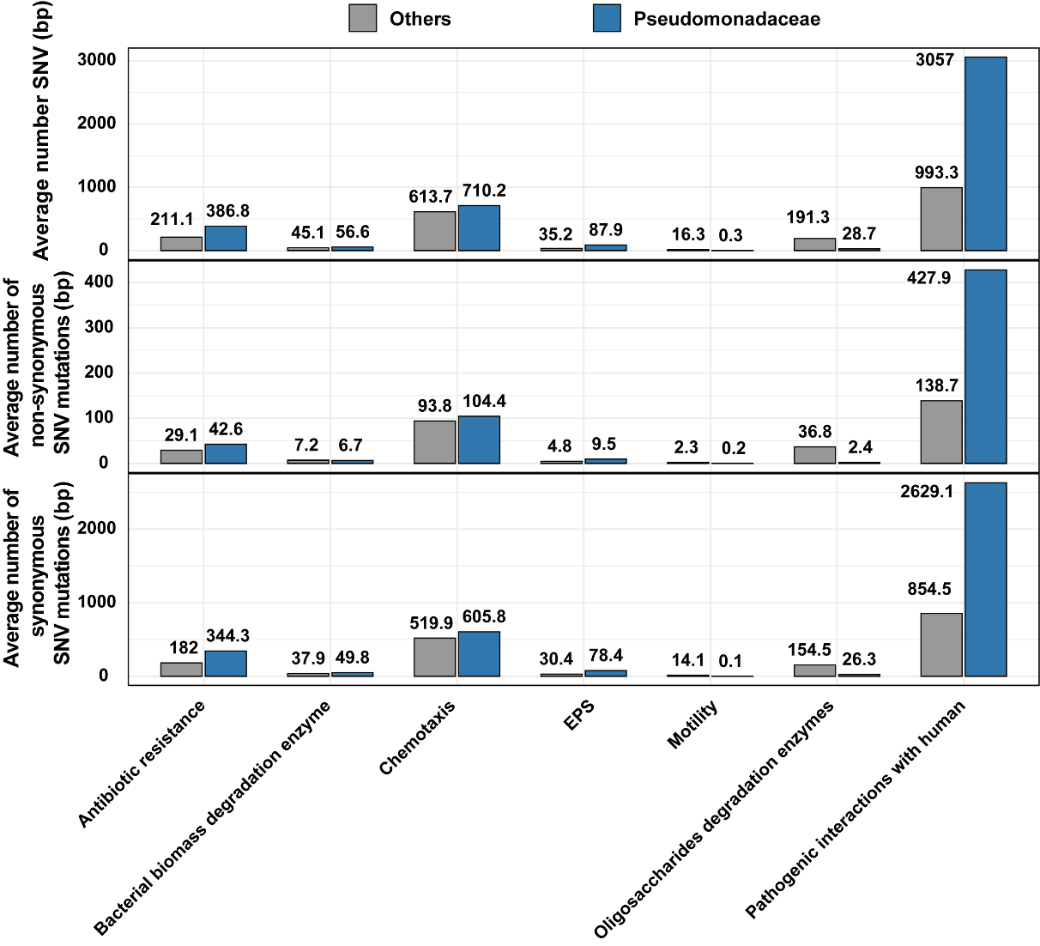


**Fig. S13. Comparison of the average SNV, synonymous, and non-synonymous mutation counts between *Pseudomonadaceae* and other bacterial families across various functional categories in cigarette butt-associated microbial communities.**

**Legends for Datasets S1-S5 (separate file)**

**Dataset S1.** Life history traits and other functional genes used in this study.

**Dataset S2.** Function of KO used in this study.

**Dataset S3.** Function of COG used in this study.

**Dataset S4.** Function of CAZy enzymes used in this study.

**Dataset S5.** Information on high-quality bacterial MAGs assembled from cigarette butts, litter, and soil samples.
