# Supplementary figures and images for: Cigarette Butts as an Emerging Urban Habitat Driving Microbial Niche Differentiation
Source: Research (Wash D C). 2026 Jul 27;9:1380. doi: 10.34133/research.1380 (PMC13402723; doi:10.34133/research.1380)

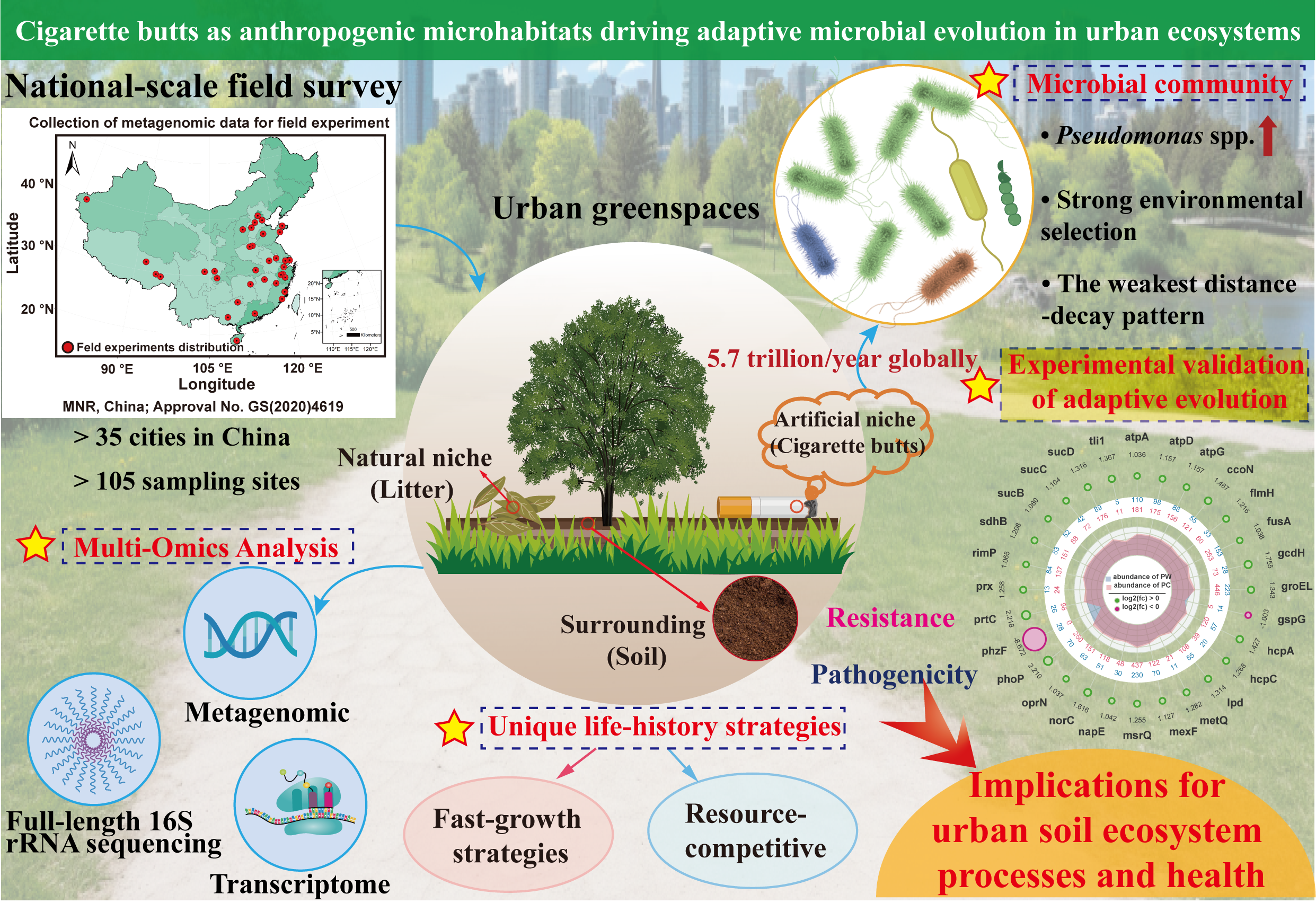

Supplement: Supplementary 1 — Graphical Abstract Figs. S1 to S13 Data S1 to S5 [file research.1380.f1.zip › Graphical Abstract.tif]
